# Three-step process for the synthesis of 10,11-dihydro-5*H*-dibenzo[*b*,*f*]azepin-10-ol derivatives†

**DOI:** 10.1039/d5ra00909j

**Published:** 2025-03-03

**Authors:** Farid M. Sroor, Thierry Terme, Patrice Vanelle, Cédric Spitz

**Affiliations:** a Organometallic and Organometalloid Chemistry Department, National Research Centre 12622 Cairo Egypt faridsroor@gmx.de; b Aix Marseille Univ., CNRS, ICR, UMR 7273, Equipe Pharmaco-Chimie Radicalaire, Faculté de Pharmacie 27 Boulevard Jean Moulin – CS 30064 Cedex 05 13385 Marseille France cedric.spitz@univ-amu.fr

## Abstract

We reported the synthesis of 10,11-dihydro-5*H*-dibenzo[*b*,*f*]azepin-10-ol derivatives, a class of compounds that has been limitedly investigated in the literature. Our approach streamlines the synthetic process, allowing straightforward access to various substituted 10,11-dihydro-5*H*-dibenzo[*b*,*f*]azepin-10-ol derivatives. This advancement not only enhances the accessibility of these derivatives for further research but also contributes to the development of potential therapeutic agents targeting various medical conditions.

## Abstract 2

We reported the synthesis of 10,11-dihydro-5*H*-dibenzo[*b*,*f*]azepin-10-ol derivatives, a class of compounds that has been limitedly investigated in the literature.

## Graphical abstract

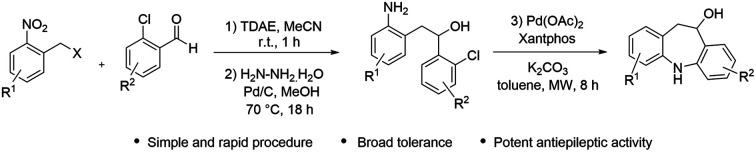

## Introduction

Dibenzazepine derivatives are a significant class of compounds in medicinal chemistry, recognized for their wide range of biological activities and therapeutic potential. The tricyclic dibenzazepine system, characterized by a fused bicyclic skeleton containing a sandwiched azepine ring between two benzene rings, displays a distinctive three-dimensional configuration that enables interactions with a wide range of biological targets.^1^ Dibenzazepine derivatives synthesis has attracted much attention. Several approaches, including cyclization reactions, ring expansion, and multicomponent reactions, are used to produce structurally different analogs efficiently.^2^

**Fig. 1:**
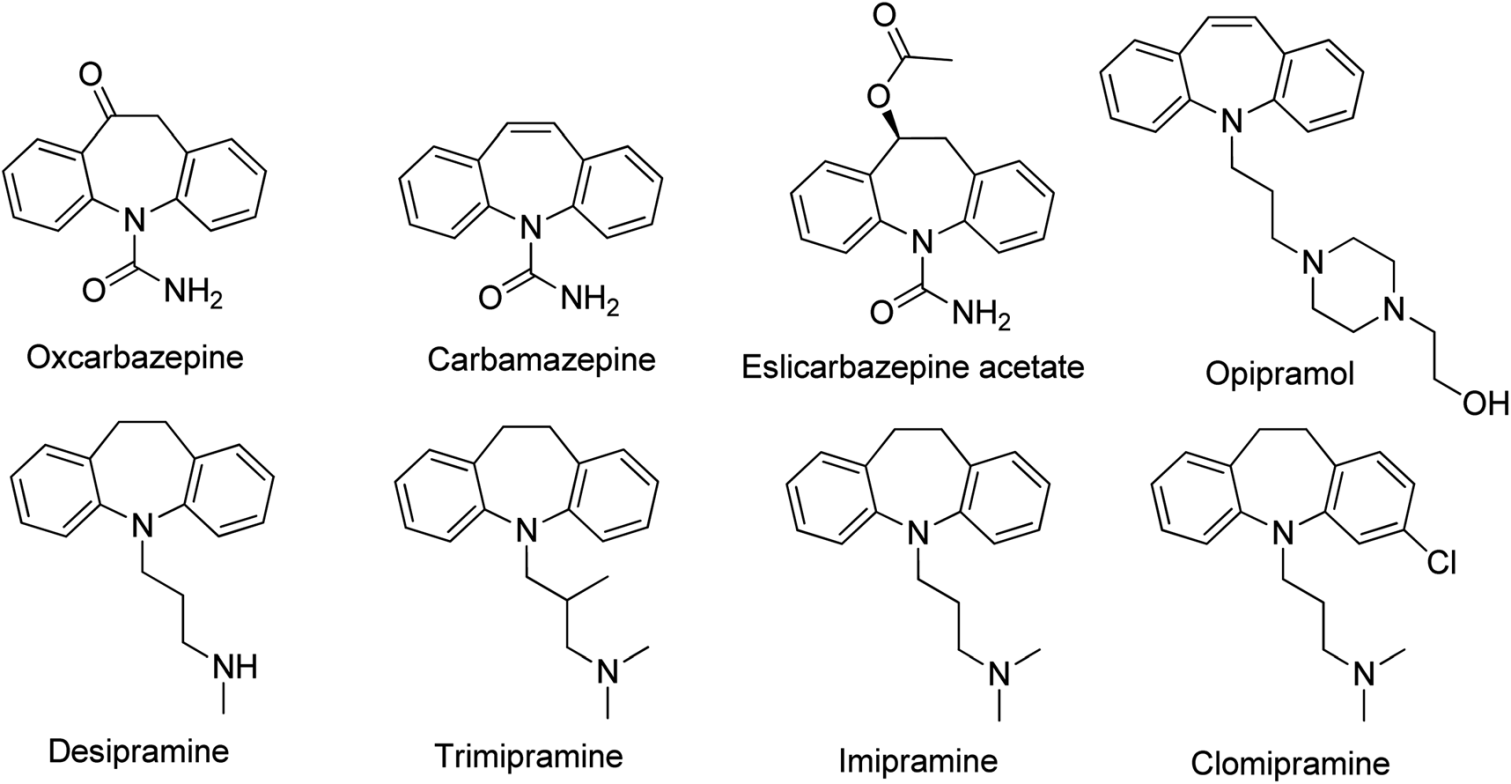
Biologically active compounds possessing dibenzazepine core.

In the same context, the dibenzo[*b*,*f*]azepine scaffold-containing compounds are known to possess an abundance of pharmacological features, making their biological activity extremely impressive, including anticancer, anti-inflammatory, antiepileptic, and antidepressant activities (Fig. 1).^3^ Their capacity to impact neurotransmitter systems, especially those in the central nervous system, has made them useful in creating medications such as novel antiepileptic drugs and mood disorders. Furthermore, certain dibenzo[*b*,*f*]azepine derivatives have shown encouraging results in blocking GPR4, suggesting their potential as a therapeutic lead for the treatment of myocardial infarction.^4^

In particular, a 10,11-dihydro-5*H*-dibenzo[*b*,*f*]azepin-10-ol derivative, eslicarbazepine acetate, was quite recently commercialized as a novel antiepileptic drug indicated for the treatment of partial-onset seizures.^5^ Eslicarbazepine acetate improves upon its predecessors, carbamazepine, and oxcarbazepine, by being available in a once-daily treatment, interacting with a reduced range of drugs, and causing lower side effects.^3*e*^ 10,11-Dihydro-5*H*-dibenzo[*b*,*f*]azepin-10-ol derivatives are usually synthesized through the ketone reduction of oxcarbazepine derivatives.^6^ Despite the critical importance of this class of heterocycles in medicinal chemistry, there is a lack of efficient and straightforward methods for their preparation. Even though the influence of the substituent on the nitrogen was already well-documented, studies on the effect of substituents on both phenyl rings are minimal. In this context, there is a need for a new process for preparing 10,11-dihydro-5*H*-dibenzo[*b*,*f*]azepin-10-ol derivatives with broad substitution patterns to modulate their biological activity. Indeed, the importance of 10,11-dihydro-5*H*-dibenzo[*b*,*f*]azepin-10-ol derivatives lies not only in their therapeutic potential but also in their role as scaffolds for exploring novel pharmacophores in the quest for new and effective treatments.

Therefore, we aim to develop a rapid and highly productive synthesis method of phenyl-substituted 10,11-dihydro-5*H*-dibenzo[*b*,*f*]azepin-10-ol derivatives.

## Results and discussion

In our endeavor, and in continuation of our previous studies to discover novel biologically active organic compounds,^7^ we established only a three-step process for the synthesis of novel 10,11-dihydro-5*H*-dibenzo[*b*,*f*]azepin-10-ol compounds from commercially available starting materials.

**Scheme 1:**
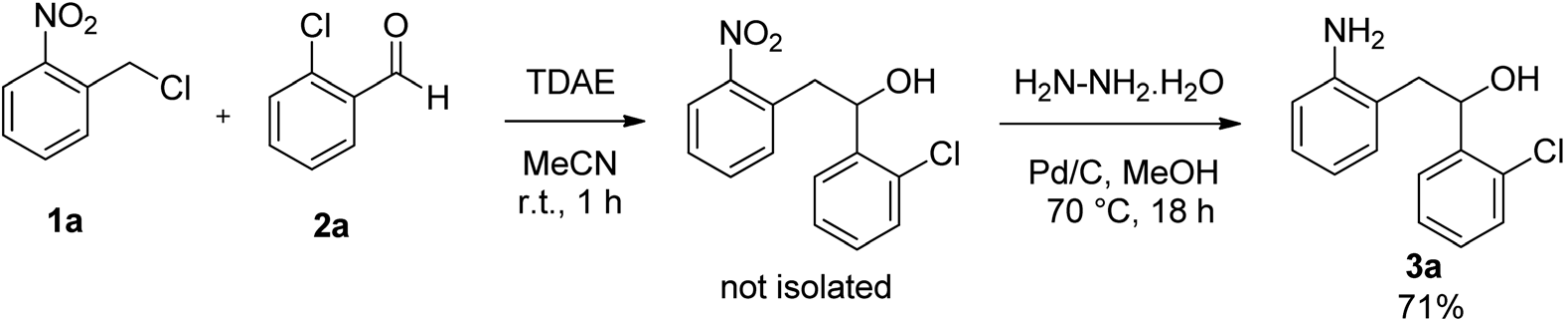
Synthesis of 2-(2-aminophenyl)-1-(2-chlorophenyl)ethanol 3a.

Tetrakis(dimethylamino)ethylene (TDAE) is an organic reducing agent, that reacts with halogenated derivatives to generate a carbanion under mild conditions.^8^ In particular, from *o*-nitrobenzyl chloride, TDAE was able to generate the corresponding *o*-nitrobenzyl carbanion, which can react with 2-chlorobenzaldehyde to allow the formation of 1-(2-chlorophenyl)-2-(2-nitrophenyl)ethanol. Then, the nitro group was reduced in the presence of Pd/C and hydrazine hydrate to afford 2-(2-aminophenyl)-1-(2-chlorophenyl)ethanol 3a in 71% overall yield (Scheme 1).

The final and more challenging step was an intramolecular Buchwald–Hartwig coupling to obtain the dibenzazepine scaffold. The reaction conditions optimization for this step was performed on 2-(2-aminophenyl)-1-(2-chlorophenyl)ethanol 3a (Table 1). In the literature, a recent patent^9^ described exactly this reaction using palladium acetate as palladium source, Xantphos as ligand, K_2_CO_3_ as base, and tetrahydrofuran as solvent at 50–60 °C for 2 h. So, we tried these conditions for the intramolecular Buchwald–Hartwig coupling of 2-(2-aminophenyl)-1-(2-chlorophenyl)ethanol 3a. Unfortunately, we obtained the dibenzazepine 4a in only 9% yield (entry 1, Table 1). No significant improvement was observed with degassed THF (entry 2). Increasing the reaction time (entry 3) and the temperature (entry 4) afforded dibenzazepine 4a in low yields. We do not have any satisfying explanation about the impossibility for us to reproduce the high yield (85–95%) described in the patent. The large scale (49.5 g, 0.2 mol of 3a) described in the patent may be more suitable for this reaction. We tried the reaction with a maximum of 5 mmol of 2-(2-aminophenyl)-1-(2-chlorophenyl)ethanol 3a but no improvement was observed. Our purpose to discover novel biologically active organic compounds was not suitable for large scale experiment. So, we performed the optimization reactions in a 0.2 mmol scale. Our studies began with screening palladium sources, ligands, bases, and solvents. Our first results (entries 5−7, Table 1) indicated that the Xphos/NaO*t*-Bu combination is unsuitable for the reaction. Using Cs_2_CO_3_ or K_2_CO_3_, poor and similar yields were obtained with a catalytic amount of Pd(OAc)_2_ and BINAP in toluene at 110 °C (entries 8 and 9). A slightly better yield was achieved using Xantphos as a ligand (entry 10). Performing the reaction under microwave irradiation and increasing the temperature allowed better yields (entries 11−13). So, the best reaction conditions for the intramolecular Buchwald–Hartwig coupling of compound 3a was the use of palladium acetate as palladium source, Xantphos as ligand, K_2_CO_3_ as base, and toluene as solvent at 170 °C under microwave irradiation for 8 h (entry 13, Table 1). As the crude NMR of compound 3a was quite clean and to save time and money, we decided not to purify intermediate 3a with column chromatography. Thus, after the two first steps, crude intermediate 3a was directly cyclized by an intramolecular Buchwald–Hartwig coupling using the previously optimized reaction conditions to give dibenzazepine 4a with a 39% overall yield (Table 2, 4a). In comparison, when intermediate 3a was purified, dibenzazepine 4a was isolated with a 37% overall yield (71% for 3a and 52% for 4a). Given this result and to save time and money, we selected the process without intermediate purification to do the scope of the reaction.

**Table 1:** Optimization of Buchwald-Hartwig coupling reaction conditions^a^

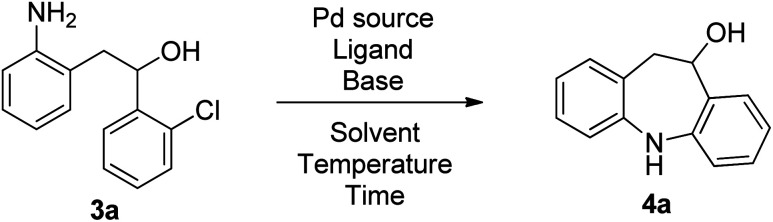							
Entry	Pd source (10 mol%)	Ligand (10 mol%)	Base (2 equiv.)	Solvent (C = 0.5 mol L^−1^)	Temperature (° C)	Time (h)	Yield^b^ (%)
1	Pd(OAc)_2_	Xantphos	K_2_CO_3_	THF	60	2	9
2	Pd(OAc)_2_	Xantphos	K_2_CO_3_	THF^c^	60	2	10
3	Pd(OAc)_2_	Xantphos	K_2_CO_3_	THF	60	16	13
4	Pd(OAc)_2_	Xantphos	K_2_CO_3_	THF	100	48	27
5	Pd_2_(dba)_3_	XPhos	NaO*t*-Bu	Toluene	110	16	<5
6	Pd_2_(dba)_3_	XPhos	NaO*t*-Bu	1,4-Dioxane	110	16	<5
7	Pd(OAc)_2_	XPhos	NaO*t*-Bu	1,4-Dioxane	110	16	<5
8	Pd(OAc)_2_	BINAP	Cs_2_CO_3_	Toluene	110	16	7
9	Pd(OAc)_2_	BINAP	K_2_CO_3_	Toluene	110	16	8
10	Pd(OAc)_2_	Xantphos	K_2_CO_3_	Toluene	110	16	14
11	Pd(OAc)_2_	Xantphos	K_2_CO_3_	Toluene	110^d^	8	16
12	Pd(OAc)_2_	Xantphos	K_2_CO_3_	Toluene	150^d^	8	31
13	Pd(OAc)_2_	Xantphos	K_2_CO_3_	Toluene	170^d^	8	52

a Reactions are performed on a 0.2 mmol scale.

b Isolated yields.

c THF was degassed with N_2_.

d Performed under microwave irradiation.

**Table 2:** Scope of the reaction^a^^,^^d^

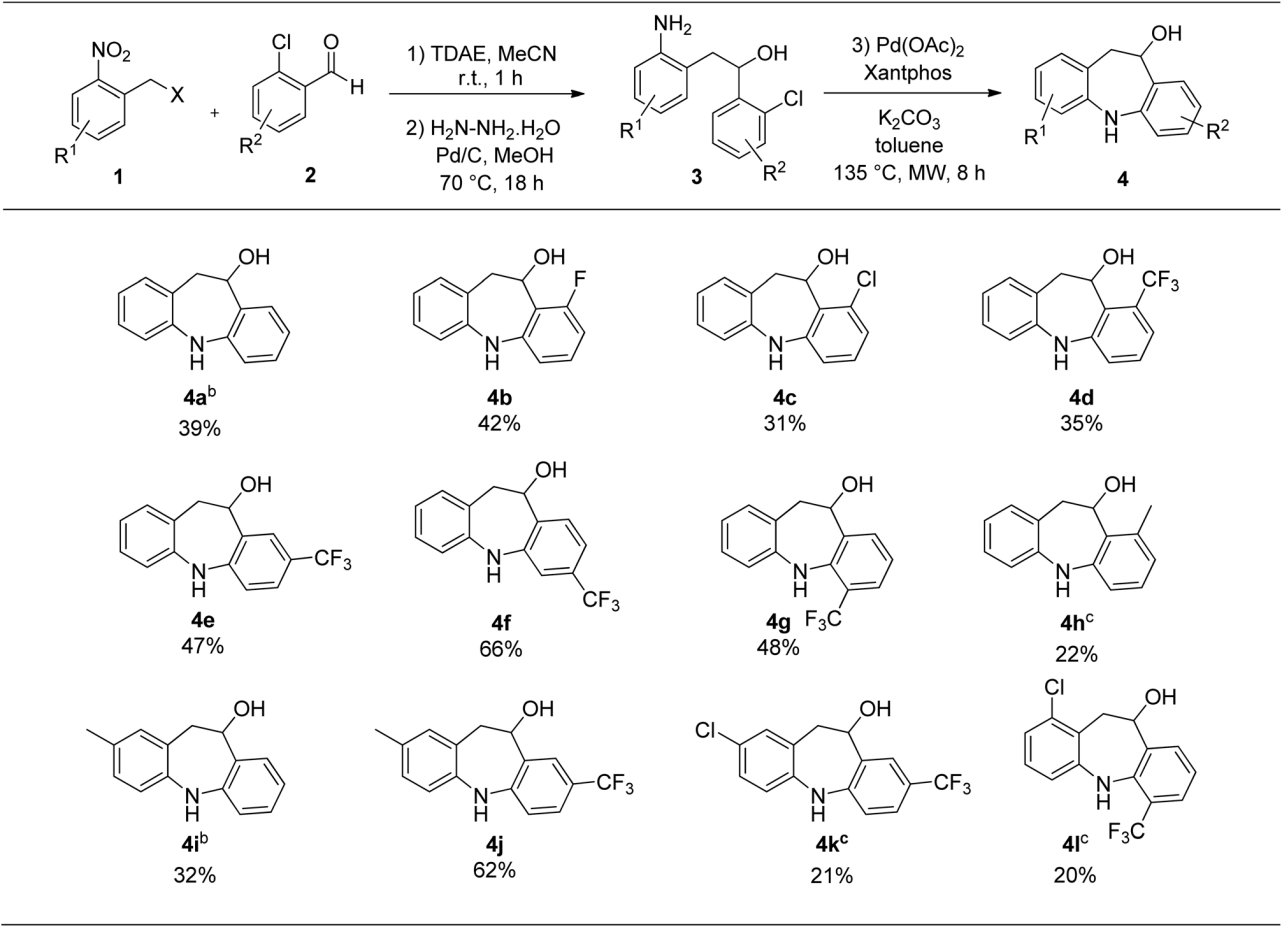

a Reaction conditions: (1) 1 (1 mmol), 2 (1.2 mmol), and TDAE (1.2 mmol) in MeCN (4 mL) were stirred at r.t. for 1 h under an inert atmosphere. (2) After evaporation of MeCN, MeOH (5 mL), Pd/C (5%, 46 mg), and hydrazine hydrate (50–60%, 312 μL) were added and the reaction was stirred at 70 °C for 18 h under an inert atmosphere. (3) Intermediate 3, Pd(OAc)_2_ (22.5 mg, 0.1 mmol, 0.1 equiv.), Xantphos (58 mg, 0.1 mmol, 0.1 equiv.), K_2_CO_3_ (276 mg, 2 mmol, 2 equiv.) and anhydrous toluene (2 mL) were stirred under microwave irradiation at 135 °C for 8 h.

b Microwave temperature for step (3) was 170 °C.

c Microwave temperature for step (3) was 150 °C.

d Isolated overall yields of the three steps are given.

**Fig. 2:**
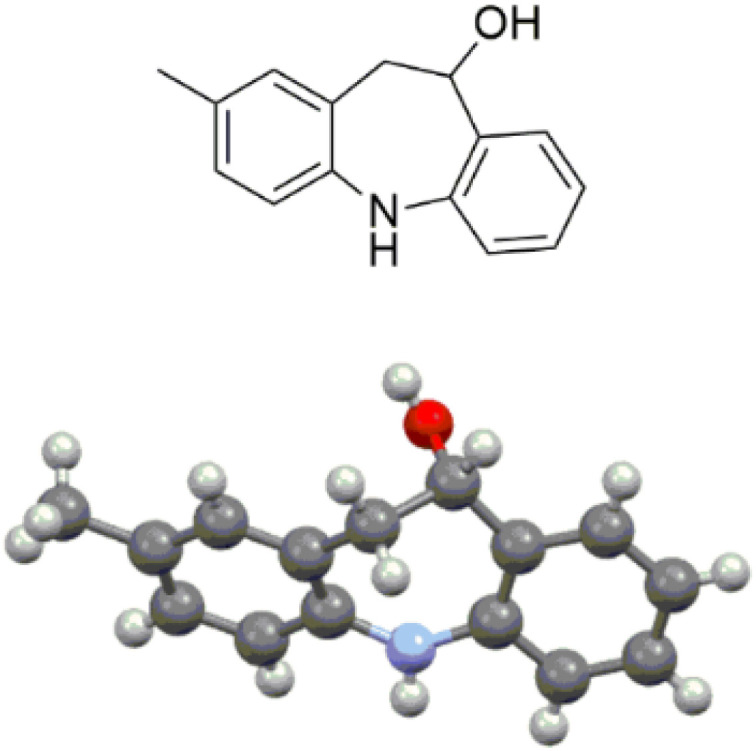
X-ray structure of dibenzazepine 4i.

Starting from di-halogenated aldehydes, dibenzazepines 4b and 4c were obtained in 42 and 31% overall yields (Table 2). Interestingly, the presence of an electron-withdrawing substituent allowed the temperature of the Buchwald–Hartwig coupling to decrease to 135 °C. As the trifluoromethyl group could enhance the biological activity, metabolic stability, lipophilicity, and pharmacokinetic properties of heterocyclic drug molecules,^10^ diversely trifluoromethyl-substituted dibenzazepines were synthesized in modest to excellent overall yields for the three-step process (Table 2, **4d–4g**). An electron-donating methyl group was well-tolerated on both aromatic parts of the dibenzazepine (Table 2, **4h–4j**). The structure of product 4i was unequivocally established by X-ray analysis (Fig. 2). Even though the reactivity was lower by introducing a chlorine atom on the left aromatic part of the dibenzazepine, compounds 4k and 4l were afforded in 21 and 20% overall yields (Table 2). Remarkably, the chlorine atom could be replaced by selected substituents, such as aromatic patterns with palladium-catalyzed cross-coupling reactions. This is a great advantage to further explore structure–activity relationship of the 10,11-dihydro-5*H*-dibenzo[*b*,*f*]azepin-10-ol scaffold.

## Conclusions

In conclusion, we have successfully reported a new, practical, and straightforward approach for synthesizing 10,11-dihydro-5*H*-dibenzo[*b*,*f*]azepin-10-ol derivatives in only three steps. Firstly, using TDAE as a mild reductant allowed the nucleophilic addition reaction of diversely substituted *o*-nitrobenzyl chlorides to different 2-chlorobenzaldehydes. Then, reduction of the nitro group afforded 2-(2-aminophenyl)-1-(2-chlorophenyl)ethanol intermediates 3. Finally, an intramolecular Buchwald–Hartwig coupling was carried out to obtain the dibenzazepine scaffold with substitution on one or both phenyl rings, contrary to the patent^9^ which only described the synthesis of dibenzazepine 4a.

## Data availability

The data supporting this article have been included as part of the ESI.† CCDC-2391789 (https://www.ccdc.cam.ac.uk/mystructures/viewinaccessstructures/39ce8207-9c8c-ef11-96ca-00505695281c) contains the supplementary crystallographic data of compound 4i for this paper. These data can be obtained free of charge from The Cambridge Crystallographic Data Centre *via*www.ccdc.cam.ac.uk/structures.

## Conflicts of interest

There are no conflicts to declare.

## Supplementary Material

RA-015-D5RA00909J-s001

RA-015-D5RA00909J-s002
