# Supplementary material for: Three-step process for the synthesis of 10,11-dihydro-5H-dibenzo[b,f]azepin-10-ol derivatives
Source: RSC Adv. 2025 Mar 3;15(9):6737–41. doi: 10.1039/d5ra00909j (PMC11875112; doi:10.1039/d5ra00909j)

Supplementary Information (SI) for RSC Advances.
This journal is © The Royal Society of Chemistry 2025

Three-step process for the synthesis of 10,11-dihydro-5*H*-dibenzo[*b*,*f*]azepin-10-ol derivatives

Farid M. Sroor,*[a] Thierry Terme,[b] Patrice Vanelle[b] and Cédric Spitz,*[b]

[a] Organometallic and Organometalloid Chemistry Department, National Research Centre, 12622 Cairo, Egypt. E-mail: faridsroor@gmx.de

[b] Aix Marseille Univ, CNRS, ICR UMR CNRS 7273, Equipe Pharmaco-Chimie Radicalaire, Faculté de Pharmacie, 27 Boulevard Jean Moulin – CS 30064 Cedex 05, 13385 Marseille, France
E-mail: cedric.spitz@univ-amu.fr

Supporting Information

General information	S2
Synthesis and characterization of 2-(2-aminophenyl)-1-(2-chlorophenyl)ethanol **3a**	S2
General procedure for the synthesis of dibenzazepines **4**	S2
Synthesis and characterization of dibenzazepines **4**	S3
References	S7
NMR spectra	S7

General information.

All reactions were carried out under a nitrogen atmosphere. All solvents and chemicals were used as purchased without further purification. Melting points were determined on a Büchi melting point B-540 apparatus and are uncorrected. HRMS analyses were performed on a Synapt G2 HDMS (Waters) with a TOF mass analyzer type at the spectropole of Aix-Marseille University. Both ^{1}H- and ^{13}C-NMR spectra were determined on a Bruker Avance NEO 400 MHz Nanobay spectrometer at the Service de RMN de la Faculté de Pharmacie de Marseille of the Aix-Marseille University. The ^{1}H and the ^{13}C chemical shifts are reported from $CDCl_3$ peaks: ^{1}H (7.26 ppm) and ^{13}C (77.16 ppm). Multiplicities are represented by the following notations: s, singlet; d, doublet; t, triplet; q, quartet; m, a more complex multiplet or overlapping multiplets. The following adsorbents were used for column chromatography: Silica gel 60 (particle size 0.063–0.200 mm, 70–230 mesh ASTM). TLC was performed on 5 x 10 cm aluminum plates coated with silica gel 60 F254 in an appropriate solvent.

Synthesis and characterization of 2-(2-aminophenyl)-1-(2-chlorophenyl)ethanol 3a.

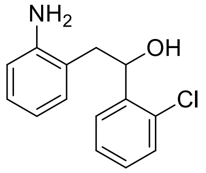

To 2-nitrobenzyl chloride **1a** (1.72 g, 10 mmol, 1 equiv.) and 2-chlorobenzaldehyde **2a** (1.43g, 12 mmol, 1.2 equiv.) in anhydrous acetonitrile (40 mL) was added TDAE (2.8 mL, 12 mmol, 1.2 equiv.) and the reaction mixture was stirred at room temperature for 1 h. The solvent was evaporated and methanol (50 mL) was added. N_2 was bubbled through methanol for 2 min, then Pd/C (5%, 460 mg) and hydrazine hydrate (50-60%, 3.12 mL) were added. The reaction mixture was stirred at 70 °C for 16 h, then filtered through a pad of celite and washed with ethyl acetate (50 mL). Brine (100 mL) was added and the phases were separated. The aqueous layer was extracted with ethyl acetate (2 x 50 mL) and the combined organic layer was dried over Na_2SO_4. Evaporation of the solvent furnished crude 2-(2-aminophenyl)-1-phenylethan-1-ol **3a**. Purification by silica gel chromatography using petroleum ether/ethyl acetate (80/20) afforded pure 2-(2-aminophenyl)-1-(2-chlorophenyl)ethanol **3a** as a white solid (1.75 g, 71%); mp 109–110 °C; ^{1}H NMR (400 MHz, $CDCl_3$) δ 7.64 (dd, *J* = 7.7, 1.7 Hz, 1H), 7.37 (dd, *J* = 7.9, 1.3 Hz, 1H), 7.33–7.29 (m, 1H), 7.25–7.21 (m, 1H), 7.11–7.06 (m, 2H), 6.78–6.72 (m, 2H), 5.36 (dd, *J* = 9.4, 2.5 Hz, 1H), 4.01 (*br*-s, 2H), 3.04 (dd, *J* = 14.4, 2.5 Hz, 1H), 2.80 (dd, *J* = 14.4, 9.4 Hz, 1H), 2.69 (*br*-s, 1H). ^{13}C NMR (101 MHz, $CDCl_3$) δ 145.5, 141.7, 131.7, 131.5, 129.5, 128.6, 128.1, 127.3, 127.1, 124.0, 119.4, 116.7, 71.9, 40.2. HRMS (ESI) : *m/z* $[M+H]^+$ calcd for $[C_{14}H_{15}ClNO]^+$: 248.0837; found : 248.0836.

General procedure for the synthesis of dibenzazepines 4.

To 2-nitrobenzyl halide **1** (1 mmol, 1 equiv.) and aldehyde **2** (1.2 mmol, 1.2 equiv.) in anhydrous acetonitrile (4 mL) was added TDAE (279 µL, 1.2 mmol, 1.2 equiv.) and the reaction mixture was stirred at room temperature for 1 h. The solvent was evaporated and methanol (5 mL) was added. N_2 was bubbled through methanol for 2 min, then Pd/C (5%, 46 mg) and hydrazine hydrate (50-60%, 312 µL) were added. The reaction mixture was stirred at 70 °C for 16 h, then filtered through a pad of celite and washed with ethyl acetate (15 mL). Brine (15 mL) was added and the phases were separated. The aqueous layer was extracted with ethyl acetate (2 x 10 mL) and the combined organic layer was dried over Na_2SO_4. Evaporation of the solvent furnished the crude 2-(2-aminophenyl)-1-phenylethan-1-ol derivatives **3**. To these crude products **3** was added palladium acetate (22.5 mg, 0.1 mmol, 0.1 equiv.), Xantphos (58 mg, 0.1 mmol, 0.1 equiv.), K_2CO_3 (276 mg, 2 mmol, 2 equiv.) and anhydrous toluene (2 mL). The reaction mixture was stirred under microwave irradiation at the appropriate temperature for 8 h, then filtered through a pad of celite and washed with ethyl acetate (15 mL). Brine (15 mL) was added and the phases were separated. The aqueous layer was extracted with ethyl acetate (2 x 10 mL) and the combined organic layer was dried over Na_2SO_4. Evaporation of the solvent furnished the crude

5*H*-dibenzo[*b,f*]azepin-10-ol derivatives **4**. Purification by silica gel chromatography afforded pure dibenzazepines **4**.

Synthesis and characterization of dibenzazepines 4.

10,11-dihydro-*5H*-dibenzo[*b,f*]azepin-10-ol **4a**

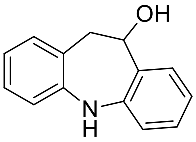

Following the general procedure with 2-nitrobenzyl chloride and 2-chlorobenzaldehyde, the intermediate **3a** was then stirred under microwave irradiation at 170 °C for 8 h. Purification by DCM/petroleum ether (from 60/40 to 100/0) afforded **4a** as a yellow solid (83 mg, 39%); ^{1}H NMR (400 MHz, $CDCl_3$) δ 7.36–7.34 (m, 1H), 7.21–7.15 (m, 3H), 6.96–6.92 (m, 1H), 6.88–6.78 (m, 3H), 6.14 (*br*-s, 1H), 5.13–5.12 (m, 1H), 3.30–3.20 (m, 2H), 1.86 (*br*-s, 1H). Spectral data match those previously reported.[1]

9-fluoro-10,11-dihydro-*5H*-dibenzo[*b,f*]azepin-10-ol **4b**

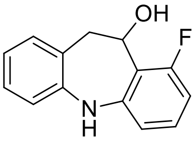

Following the general procedure with 2-nitrobenzyl chloride and 2-chloro-6-fluorobenzaldehyde, the intermediate **3b** was then stirred under microwave irradiation at 135 °C for 8 h. Purification by DCM/petroleum ether (from 60/40 to 100/0) afforded **4b** as a beige solid (96 mg, 42%); mp 139–141 °C; ^{1}H NMR (400 MHz, $CDCl_3$) δ 7.26–7.24 (m, 1H), 7.21–7.16 (m, 1H), 7.12–7.07 (m, 1H), 7.01–6.97 (m, 1H), 6.86 (dd, *J* = 7.9, 0.8 Hz, 1H), 6.59–6.56 (m, 2H), 6.16 (*br*-s, 1H), 5.61–5.59 (m, 1H), 3.25–3.18 (m, 2H), 1.75 (d, *J* = 7.7 Hz, 1H). ^{13}C NMR (101 MHz, $CDCl_3$) δ 163.0 (d, *J* = 245.4 Hz), 144.2 (d, *J* = 6.1 Hz), 142.9, 132.5, 129.3 (d, *J* = 11.1 Hz), 127.8, 125.7, 122.4, 118.7, 116.4 (d, *J* = 18.2 Hz), 114.2 (d, *J* = 3.0 Hz), 105.8 (d, *J* = 24.2 Hz), 63.8 (d, *J* = 7.1 Hz), 39.4. HRMS (ESI) : *m/z* $[M+Na]^+$ calcd for $[C_{14}H_{12}FNONa]^+$: 252.0795; found : 252.0798.

9-chloro-10,11-dihydro-*5H*-dibenzo[*b,f*]azepin-10-ol **4c**

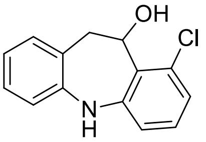

Following the general procedure with 2-nitrobenzyl chloride and 2,6-dichlorobenzaldehyde, the intermediate **3c** was then stirred under microwave irradiation at 135 °C for 8 h. Purification by DCM/petroleum ether (from 60/40 to 100/0) afforded **4c** as a beige solid (76 mg, 31%); mp 143–145 °C; ^{1}H NMR (400 MHz, $CDCl_3$) δ 7.26–7.24 (m, 1H), 7.20–7.16 (m, 1H), 7.08–7.04 (m, 1H), 7.02–6.98 (m, 1H), 6.92 (dd, *J* = 7.8, 1.2 Hz, 1H), 6.85 (dd, *J* = 7.9, 0.8 Hz, 1H), 6.71 (dd, *J* = 8.2, 1.0 Hz, 1H), 6.10 (*br*-s, 1H), 5.74–5.70 (m, 1H), 3.24–3.22 (m, 2H), 2.02 (d, *J* = 7.1 Hz, 1H). ^{13}C NMR (101 MHz, $CDCl_3$) δ 144.5, 142.3, 137.0, 132.2, 129.0, 127.7, 125.9, 125.4, 122.4, 120.8, 118.6, 118.0, 68.0, 39.1. HRMS (ESI) : *m/z* $[M+Na]^+$ calcd for $[C_{14}H_{12}ClNONa]^+$: 268.0500; found : 248.0497.

9-(trifluoromethyl)-10,11-dihydro-*5H*-dibenzo[*b*,*f*]azepin-10-ol **4d**

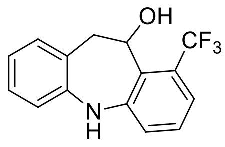

Following the general procedure with 2-nitrobenzyl chloride and 2-chloro-6-(trifluoromethyl)benzaldehyde, the intermediate **3d** was then stirred under microwave irradiation at 135 °C for 8 h. Purification by DCM/petroleum ether (from 60/40 to 100/0) afforded **4d** as a yellow solid (98 mg, 35%); mp 148–149 °C; ^{1}H NMR (400 MHz, $CDCl_3$) δ 7.23–7.21 (m, 2H), 7.19–7.15 (m, 2H), 7.03–7.01 (m, 1H), 6.96–6.92 (m, 1H), 6.83 (dd, *J* = 8.4, 1.0 Hz, 1H), 6.30 (s, 1H), 5.65–5.62 (m, 1H), 3.40 (dd, *J* = 15.4, 6.9 Hz, 1H), 3.24–3.21 (m, 1H), 2.54 (d, *J* = 7.3 Hz, 1H). ^{13}C NMR (101 MHz, $CDCl_3$) δ 144.0, 141.2, 132.8, 129.7 (q, *J* = 29.3 Hz), 128.3, 127.9, 127.7, 124.7 (q, *J* = 275.7 Hz), 123.7, 123.4, 121.6, 118.2, 117.8 (q, *J* = 6.4 Hz), 65.8 (q, *J* = 2.9 Hz), 40.0. HRMS (ESI) : *m/z* $[M+Na]^+$ calcd for $[C_{15}H_{12}F_3NONa]^+$: 302.0763; found : 302.0761.

8-(trifluoromethyl)-10,11-dihydro-*5H*-dibenzo[*b*,*f*]azepin-10-ol **4e**

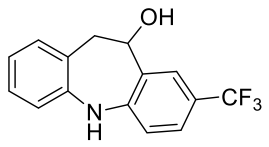

Following the general procedure with 2-nitrobenzyl chloride and 2-chloro-5-(trifluoromethyl)benzaldehyde, the intermediate **3e** was then stirred under microwave irradiation at 135 °C for 8 h. Purification by DCM/petroleum ether (from 60/40 to 100/0) afforded **4e** as a beige solid (131 mg, 47%); mp 143–144 °C; ^{1}H NMR (400 MHz, $CDCl_3$) δ 7.63 (d, *J* = 1.3 Hz, 1H), 7.39 (dd, *J* = 8.5, 1.9 Hz, 1H), 7.22–7.17 (m, 2H), 7.00–6.96 (m, 1H), 6.86–6.82 (m, 2H), 6.36 (*br*-s, 1H), 5.14–5.10 (m, 1H), 3.30–3.22 (m, 2H), 1.76 (d, *J* = 7.5 Hz, 1H). ^{13}C NMR (101 MHz, $CDCl_3$) δ 144.4, 141.6, 132.5, 128.9 (q, *J* = 4.0 Hz), 128.00, 127.96, 125.6 (q, *J* = 3.6 Hz), 124.6 (q, *J* = 271.7 Hz), 124.2, 122.2, 121.0 (q, *J* = 33.3 Hz), 118.64, 118.62, 71.5, 41.1. HRMS (ESI) : *m/z* $[M+Na]^+$ calcd for $[C_{15}H_{12}F_3NONa]^+$: 302.0763; found : 302.0765.

7-(trifluoromethyl)-10,11-dihydro-*5H*-dibenzo[*b*,*f*]azepin-10-ol **4f**

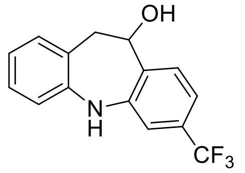

Following the general procedure with 2-nitrobenzyl chloride and 2-chloro-4-(trifluoromethyl)benzaldehyde, the intermediate **3f** was then stirred under microwave irradiation at 135 °C for 8 h. Purification by DCM/petroleum ether (from 60/40 to 100/0) afforded **4f** as a beige solid (184 mg, 66%); mp 182–184 °C; ^{1}H NMR (400 MHz, $CDCl_3$) δ 7.48 (d, *J* = 7.8 Hz, 1H), 7.21–7.17 (m, 2H), 7.08–7.06 (m, 2H), 6.99–6.95 (m, 1H), 6.85 (d, *J* = 7.9 Hz, 1H), 6.23 (*br*-s, 1H), 5.17–5.12 (m, 1H), 3.27–3.26 (m, 2H), 1.74 (d, *J* = 8.0 Hz, 1H). ^{13}C NMR (101 MHz, $CDCl_3$) δ 142.2, 142.0, 132.5, 132.1, 131.8, 131.0 (q, *J* = 32.3 Hz), 128.0, 124.1, 124.0 (q, *J* = 273.7 Hz), 122.1, 118.6, 115.8 (q, *J* = 3.6 Hz), 115.4 (q, *J* = 4.0 Hz), 71.3, 41.0. HRMS (ESI) : *m/z* $[M+Na]^+$ calcd for $[C_{15}H_{12}F_3NONa]^+$: 302.0763; found : 302.0762.

6-(trifluoromethyl)-10,11-dihydro-*5H*-dibenzo[*b*,*f*]azepin-10-ol **4g**

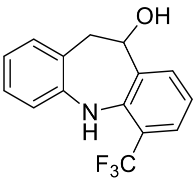

Following the general procedure with 2-nitrobenzyl chloride and 2-chloro-3-(trifluoromethyl)benzaldehyde, the intermediate **3g** was then stirred under microwave irradiation at 135 °C for 8 h. Purification by DCM/petroleum ether (from 60/40 to 100/0) afforded **4g** as a beige solid (134 mg, 48%); mp 118–119 °C; ^{1}H NMR (400 MHz, $CDCl_3$) δ 7.56 (d, *J* = 7.6 Hz, 1H), 7.51 (dd, *J* = 7.8, 1.2 Hz, 1H), 7.20–7.16 (m, 2H), 6.99–6.94 (m, 2H), 6.87 (d, *J* = 8.1 Hz, 1H), 6.43 (*br*-s, 1H), 5.23–5.20 (m, 1H), 3.34 (dd, *J* = 14.8, 2.1 Hz, 1H), 3.26 (dd, *J* = 14.8, 7.6 Hz, 1H), 2.24 (d, *J* = 6.9 Hz, 1H). ^{13}C NMR (101 MHz, $CDCl_3$) δ 141.8, 140.3 (q, *J* = 1.2 Hz), 133.8, 133.4, 132.2, 127.8, 126.3 (q, *J* = 6.1 Hz), 125.0 (q, *J* = 273.7 Hz), 124.7, 122.3, 119.8, 119.7, 117.8 (q, *J* = 28.3 Hz), 71.5, 40.9. HRMS (ESI) : *m/z* $[M+Na]^+$ calcd for $[C_{15}H_{12}F_3NONa]^+$: 302.0763; found : 302.0763.

9-methyl-10,11-dihydro-*5H*-dibenzo[*b*,*f*]azepin-10-ol **4h**

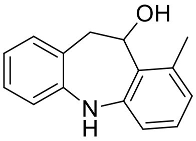

Following the general procedure with 2-nitrobenzyl chloride and 2-chloro-6-methylbenzaldehyde, the intermediate **3h** was then stirred under microwave irradiation at 150 °C for 8 h. Purification by DCM/petroleum ether (from 60/40 to 100/0) afforded **4h** as a white solid (50 mg, 22%); mp 157–159 °C; ^{1}H NMR (400 MHz, $CDCl_3$) δ 7.23 (d, *J* = 7.4 Hz, 1H), 7.18–7.14 (m, 1H), 7.07–7.03 (m, 1H), 6.98–6.94 (m, 1H), 6.83 (dd, *J* = 7.9, 0.8 Hz, 1H), 6.73 (d, *J* = 7.4 Hz, 1H), 6.66 (d, *J* = 8.1 Hz, 1H), 5.96 (*br*-s, 1H), 5.40 (ddd, *J* = 9.5, 6.3, 1.2 Hz, 1H), 3.29 (d, *J* = 14.0 Hz, 1H), 3.19 (dd, *J* = 14.0, 6.3 Hz, 1H), 2.48 (s, 3H), 1.62 (d, *J* = 9.5 Hz, 1H). ^{13}C NMR (101 MHz, $CDCl_3$) δ 143.8, 143.1, 140.0, 132.3, 128.4, 127.7, 126.9, 125.7, 122.3, 121.9, 118.5, 117.5, 67.6, 39.7, 20.3. HRMS (ESI) : *m/z* $[M+Na]^+$ calcd for $[C_{15}H_{15}NONa]^+$: 248.1046; found : 248.1049.

2-methyl-10,11-dihydro-*5H*-dibenzo[*b*,*f*]azepin-10-ol **4i**

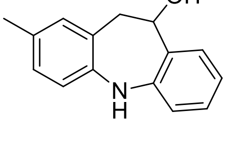

Following the general procedure with 5-methyl-2-nitrobenzyl chloride and 2-chlorobenzaldehyde, the intermediate **3i** was then stirred under microwave irradiation at 170 °C for 8 h. Purification by DCM/petroleum ether (from 60/40 to 100/0) afforded **4i** as a yellow solid (72 mg, 32%); mp 136–138 °C; ^{1}H NMR (400 MHz, $CDCl_3$) δ 7.34 (dd, *J* = 7.6, 1.4 Hz, 1H), 7.16 (ddd, *J* = 8.0, 7.3, 1.6 Hz, 1H), 7.01 (s, 1H), 6.98–6.95 (m, 1H), 6.85–6.81 (m, 1H), 6.77 (dd, *J* = 8.1, 1.0 Hz, 1H), 6.73 (d, *J* = 8.0 Hz, 1H), 6.02 (*br*-s, 1H), 5.12 (d, *J* = 6.4 Hz, 1H), 3.28–3.16 (m, 2H), 2.29 (s, 3H), 1.72 (*br*-s, 1H). ^{13}C NMR (101 MHz, $CDCl_3$) δ 142.2, 140.6, 133.0, 131.9, 130.9, 128.7, 128.30, 128.27, 124.4, 119.3, 118.5, 118.3, 71.9, 40.7, 20.6. HRMS (ESI) : *m/z* $[M+Na]^+$ calcd for $[C_{15}H_{15}NONa]^+$: 248.1046; found : 248.1047.

2-methyl-8-(trifluoromethyl)-10,11-dihydro-*5H*-dibenzo[*b,f*]azepin-10-ol **4j**

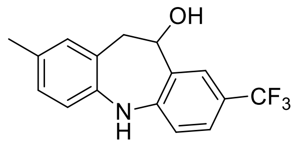

Following the general procedure with 5-methyl-2-nitrobenzyl chloride and 2-chloro-5-(trifluoromethyl)benzaldehyde, the intermediate **3j** was then stirred under microwave irradiation at 135 °C for 8 h. Purification by DCM/petroleum ether (from 60/40 to 100/0) afforded **4j** as a beige solid (182 mg, 62%); mp 156–157 °C; ^{1}H NMR (400 MHz, $CDCl_3$) δ 7.61 (d, *J* = 1.3 Hz, 1H), 7.37 (dd, *J* = 8.5, 1.8 Hz, 1H), 7.02–6.99 (m, 2H), 6.81 (d, *J* = 8.5 Hz, 1H), 6.76 (d, *J* = 7.9 Hz, 1H), 6.30 (*br*-s, 1H), 5.14–5.10 (m, 1H), 3.22–3.21 (m, 2H), 2.30 (s, 3H), 1.74 (d, *J* = 7.6 Hz, 1H). ^{13}C NMR (101 MHz, $CDCl_3$) δ 144.7, 139.2, 133.0, 131.8, 129.1 (q, *J* = 3.8 Hz), 128.5, 127.6, 125.6 (q, *J* = 3.6 Hz), 124.7 (q, *J* = 271.7 Hz), 124.2, 120.6 (q, *J* = 33.3 Hz), 118.6, 118.4, 71.6, 40.9, 20.6. HRMS (ESI) : *m/z* $[M+Na]^+$ calcd for $[C_{16}H_{14}F_3NONa]^+$: 316.0920; found : 316.0918.

2-chloro-8-(trifluoromethyl)-10,11-dihydro-*5H*-dibenzo[*b,f*]azepin-10-ol **4k**

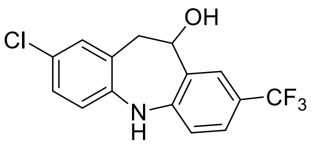

Following the general procedure with 5-chloro-2-nitrobenzyl bromide and 2-chloro-5-(trifluoromethyl)benzaldehyde, the intermediate **3k** was then stirred under microwave irradiation at 150 °C for 8 h. Purification by DCM/petroleum ether (from 60/40 to 100/0) afforded **4k** as a beige solid (66 mg, 21%); mp 128–130 °C; ^{1}H NMR (400 MHz, $CDCl_3$) δ 7.63 (d, *J* = 1.4 Hz, 1H), 7.40 (dd, *J* = 8.4, 1.8 Hz, 1H), 7.19 (d, *J* = 2.4 Hz, 1H), 7.15 (dd, *J* = 8.4, 2.4 Hz, 1H), 6.84 (d, *J* = 8.4 Hz, 1H), 6.79 (d, *J* = 8.5 Hz, 1H), 6.33 (*br*-s, 1H), 5.15–5.12 (m, 1H), 3.27–3.19 (m, 2H), 1.76 (d, *J* = 6.7 Hz, 1H). ^{13}C NMR (101 MHz, $CDCl_3$) δ 144.0, 140.2, 132.0, 128.8 (q, *J* = 4.0 Hz), 127.9, 127.8, 127.0, 126.1, 125.9 (q, *J* = 3.0 Hz), 124.5 (q, *J* = 272.2 Hz), 121.4 (q, *J* = 32.3 Hz), 119.8, 118.7, 71.3, 40.9. HRMS (ESI) : *m/z* $[M+Na]^+$ calcd for $[C_{15}H_{11}ClF_3NONa]^+$: 336.0373; found : 336.0372.

1-chloro-6-(trifluoromethyl)-10,11-dihydro-*5H*-dibenzo[*b,f*]azepin-10-ol **4l**

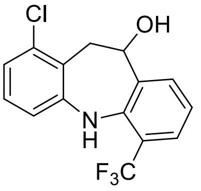

Following the general procedure with 6-chloro-2-nitrobenzyl chloride and 2-chloro-3-(trifluoromethyl)benzaldehyde, the intermediate **3l** was then stirred under microwave irradiation at 150 °C for 8 h. Purification by DCM/petroleum ether (from 60/40 to 100/0) afforded **4l** as a beige solid (63 mg, 20%); mp 111–113 °C; ^{1}H NMR (400 MHz, $CDCl_3$) δ 7.66 (d, *J* = 7.6 Hz, 1H), 7.51 (d, *J* = 7.7 Hz, 1H), 7.10–7.05 (m, 3H), 6.80– 6.76 (m, 1H), 6.19 (*br*-s, 1H), 5.40–5.33 (m, 1H), 3.46–3.37 (m, 2H), 2.48 (*br*-s, 1H). ^{13}C NMR (101 MHz, $CDCl_3$) δ 143.8, 140.2 (q, *J* = 2.0 Hz), 136.5, 135.7, 132.0, 127.9, 125.9 (q, *J* = 6.1 Hz), 124.8 (q, *J* = 273.7 Hz), 123.7, 123.5, 121.6, 119.1 (q, *J* = 29.3 Hz), 118.6, 70.1, 38.4. HRMS (ESI) : *m/z* $[M+Na]^+$ calcd for $[C_{15}H_{11}ClF_3NONa]^+$: 336.0373; found : 336.0374.

References

[1] C. Yin, X.-Q. Dong, X. Zhang, *Adv. Synth. Catal.* **2018**, *360*, 4319–4324.

NMR spectra

2-(2-aminophenyl)-1-(2-chlorophenyl)ethanol **3a**

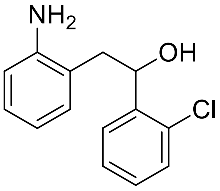

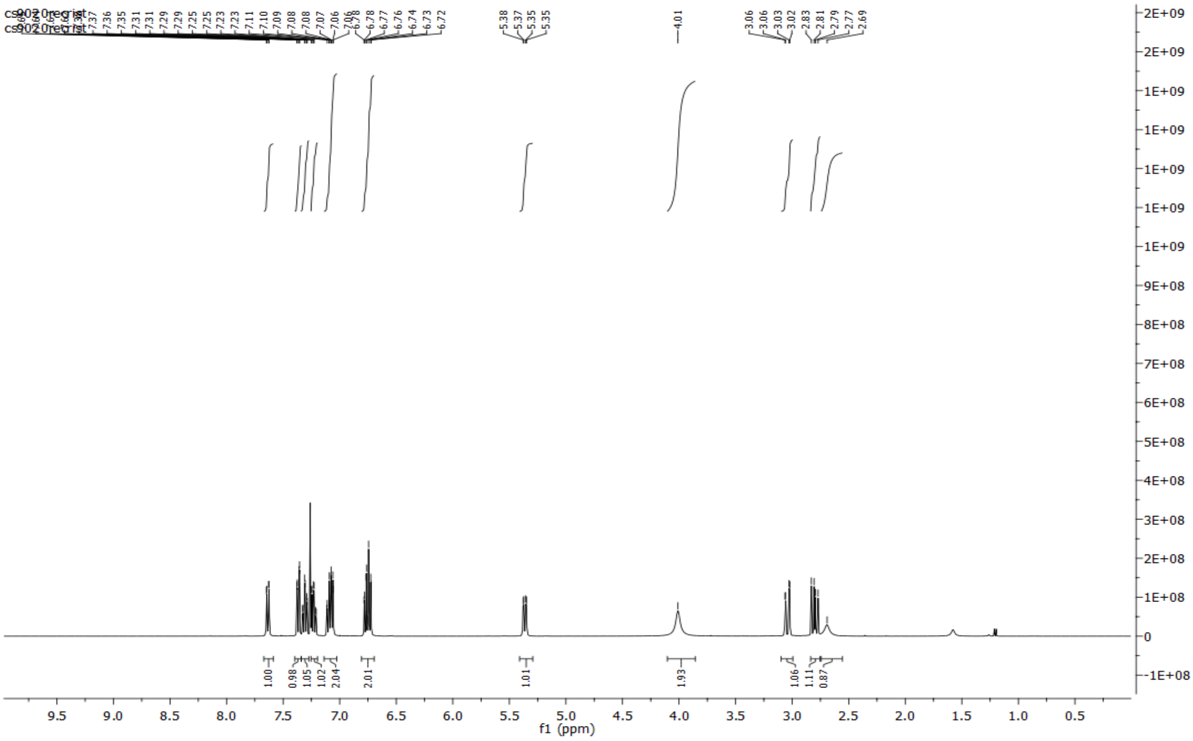

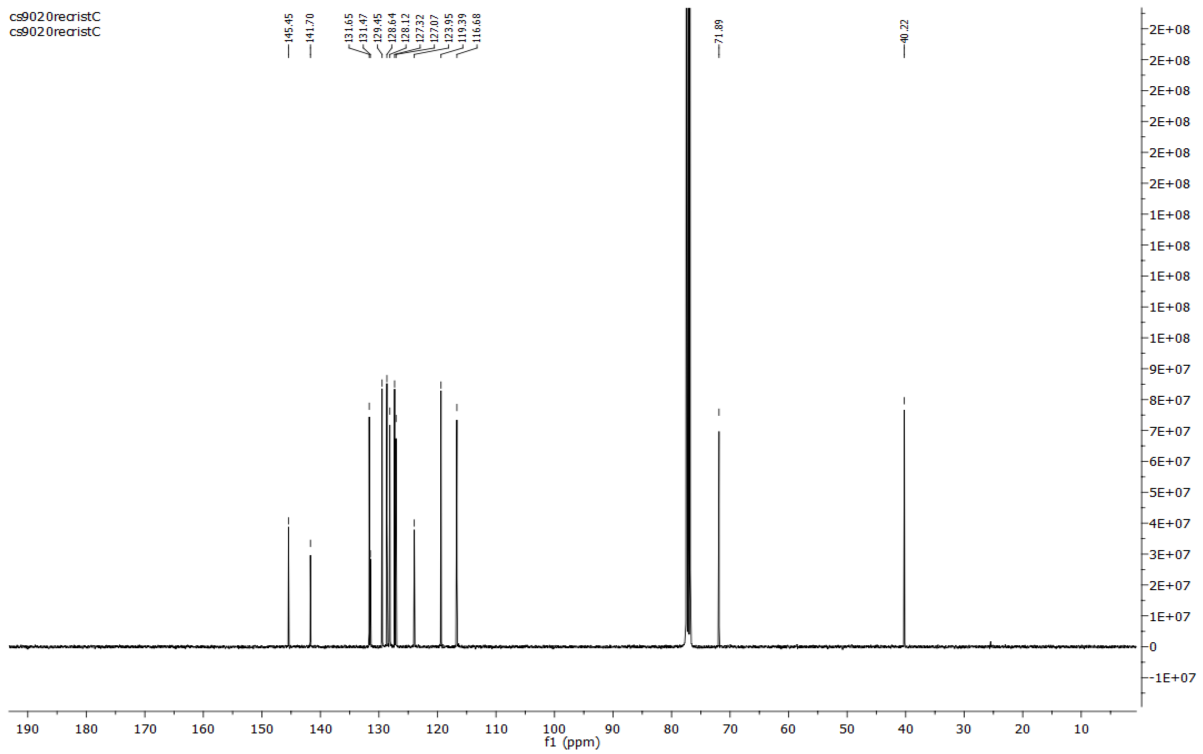

10,11-dihydro-*5H*-dibenzo[*b*,*f*]azepin-10-ol **4a**

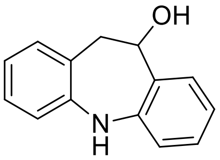

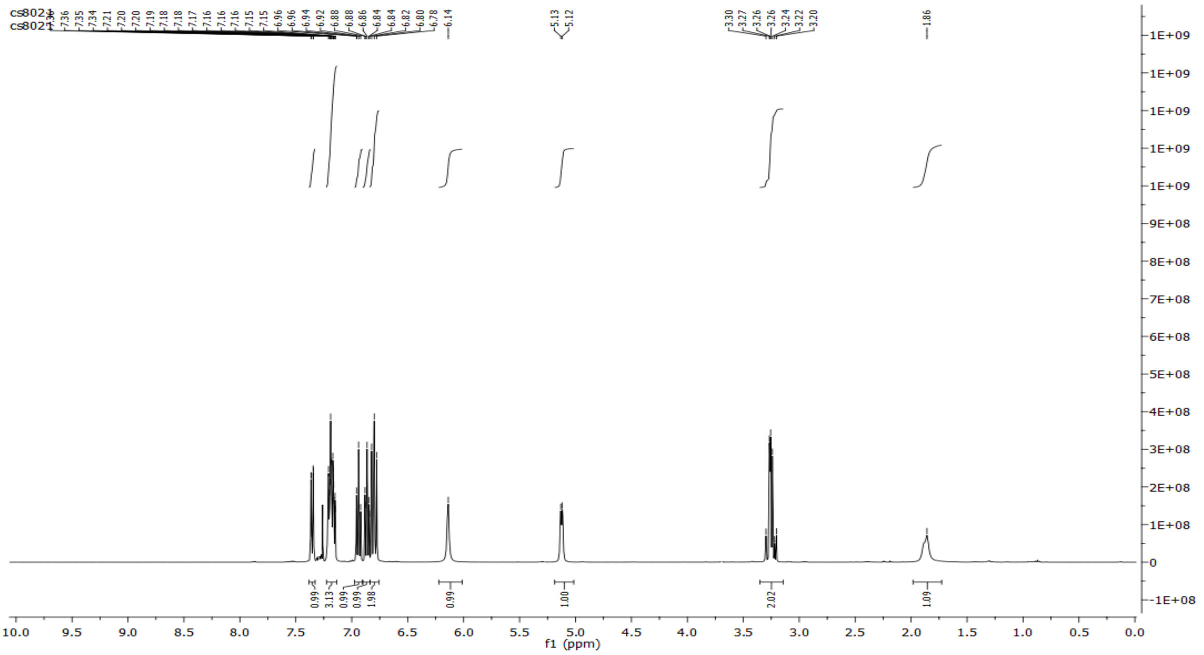

9-fluoro-10,11-dihydro-*5H*-dibenzo[*b*,*f*]azepin-10-ol **4b**

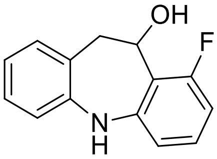

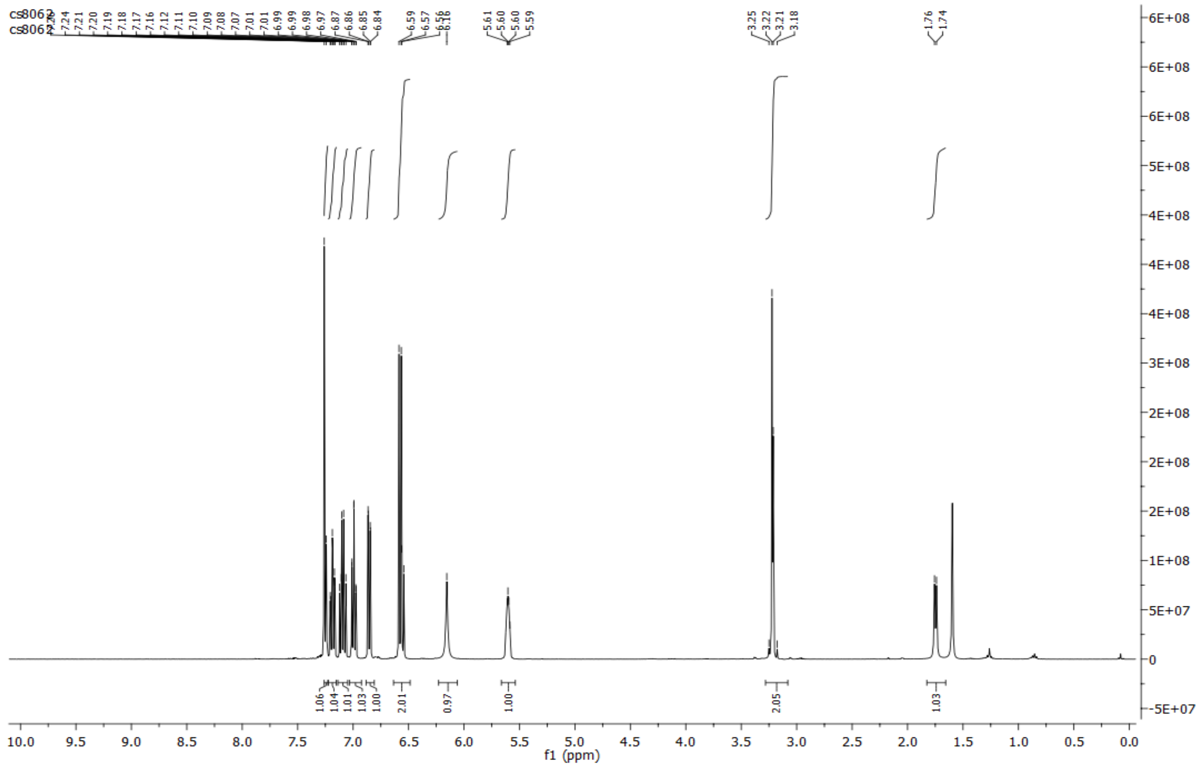

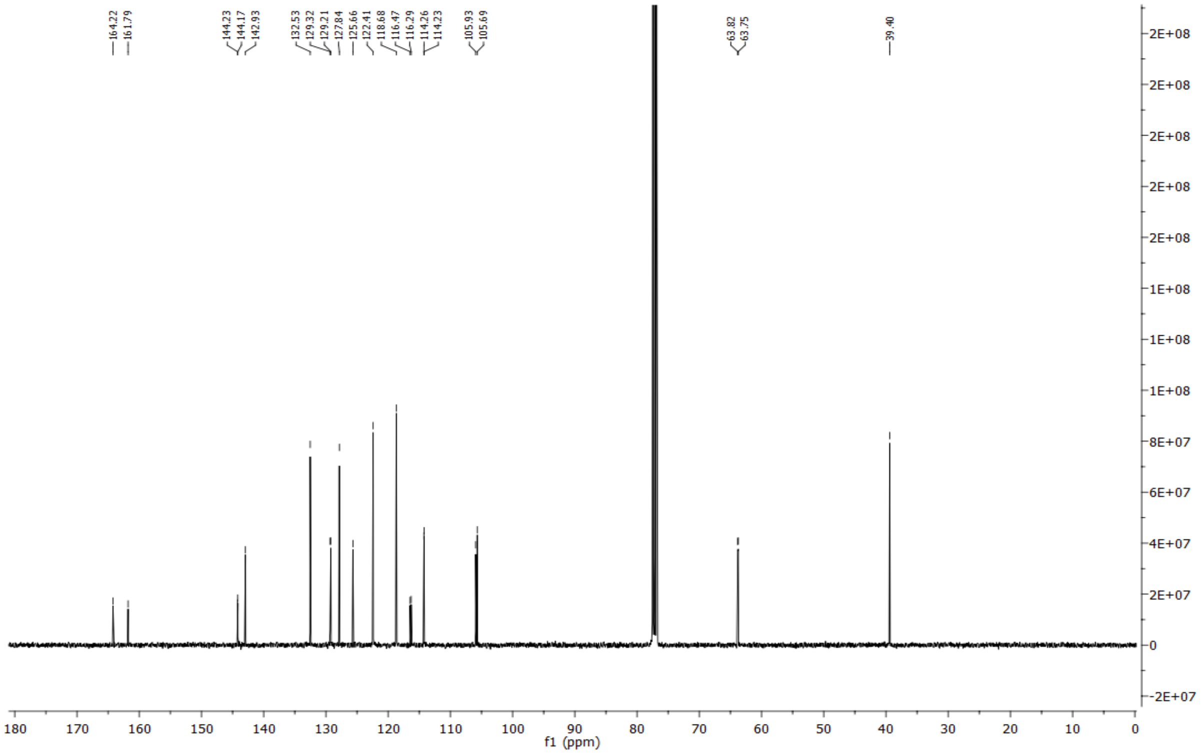

9-chloro-10,11-dihydro-*5H*-dibenzo[*b*,*f*]azepin-10-ol **4c**

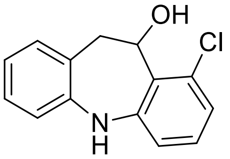

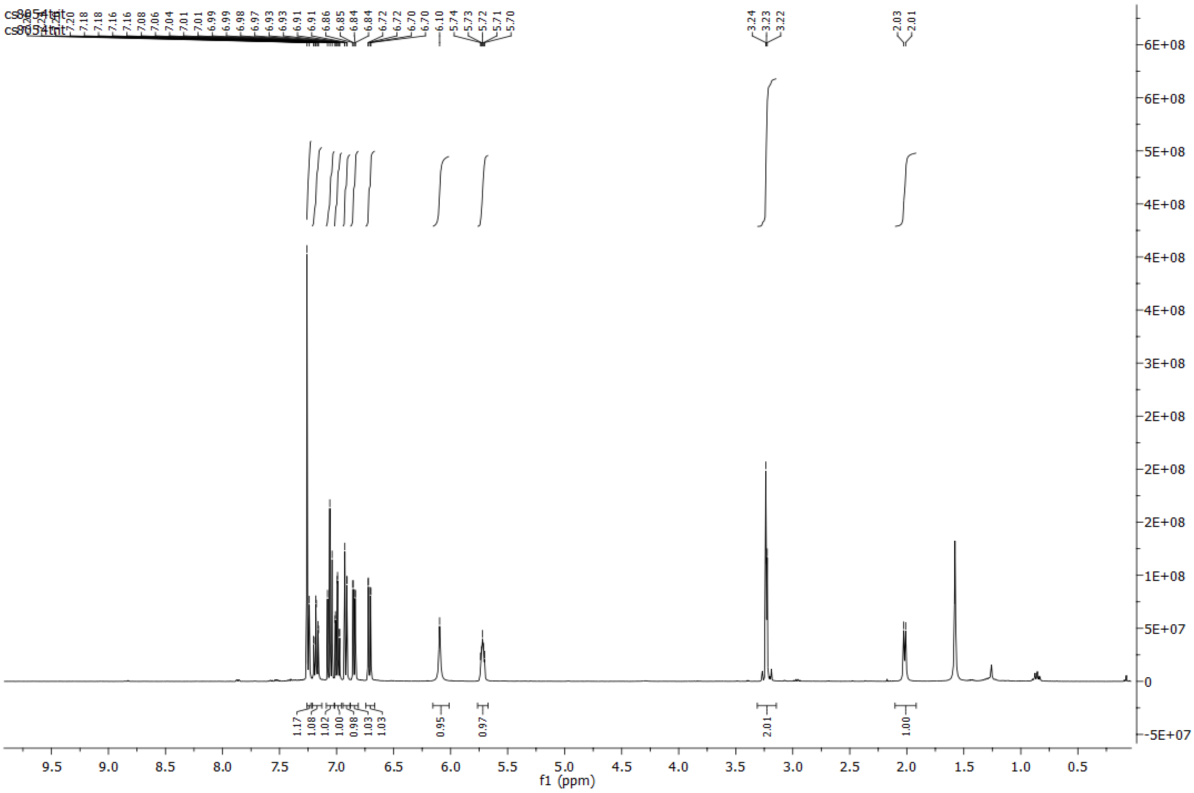

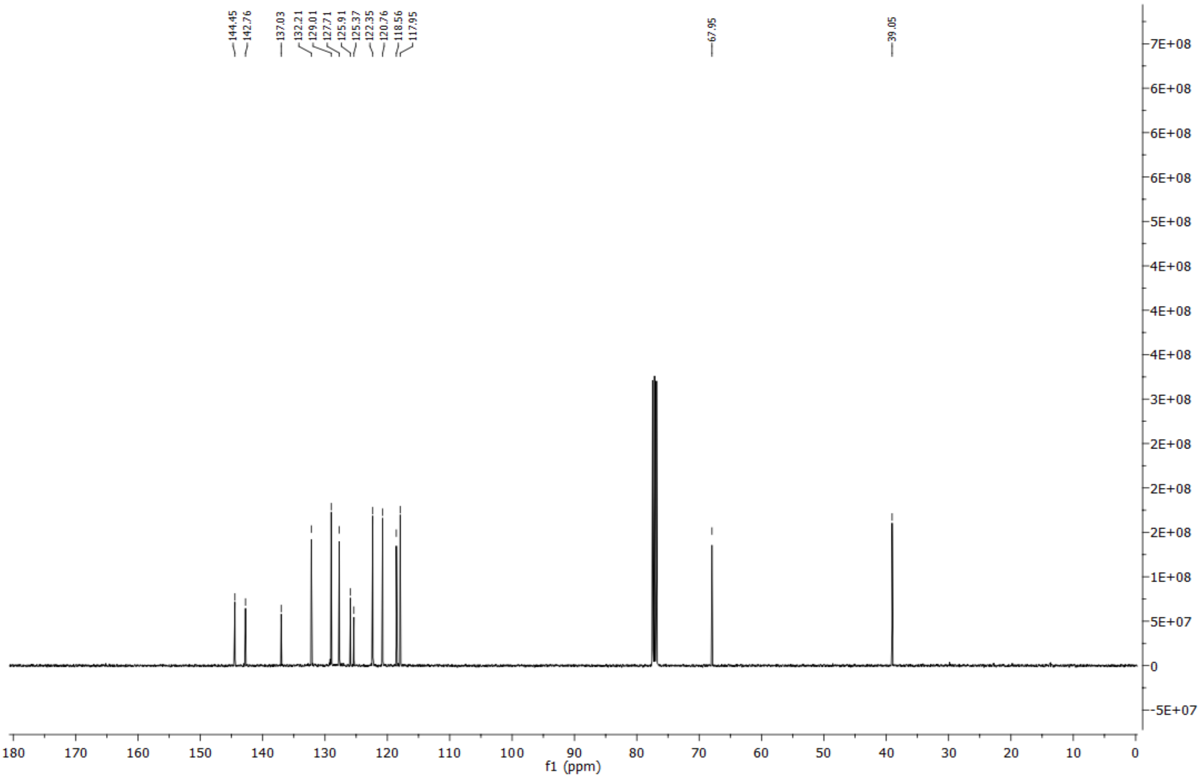

9-(trifluoromethyl)-10,11-dihydro-*5H*-dibenzo[*b*,*f*]azepin-10-ol **4d**

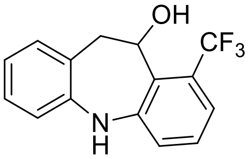

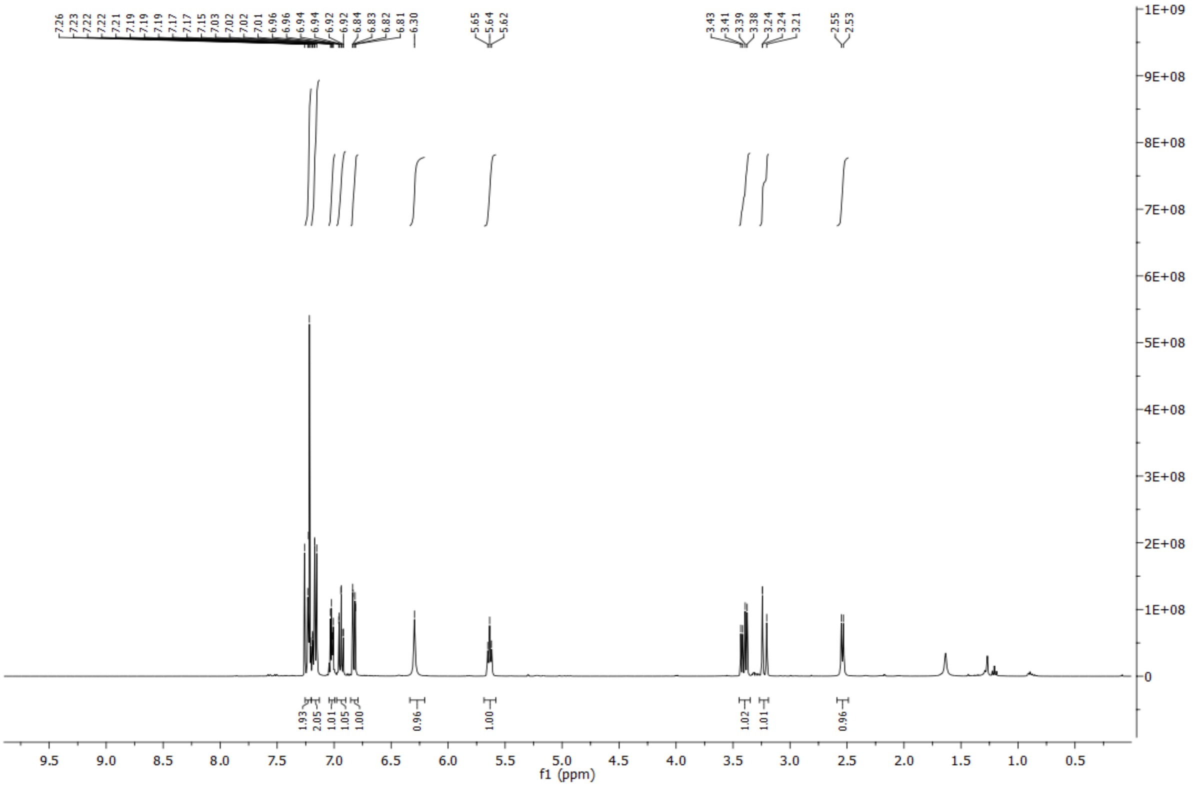

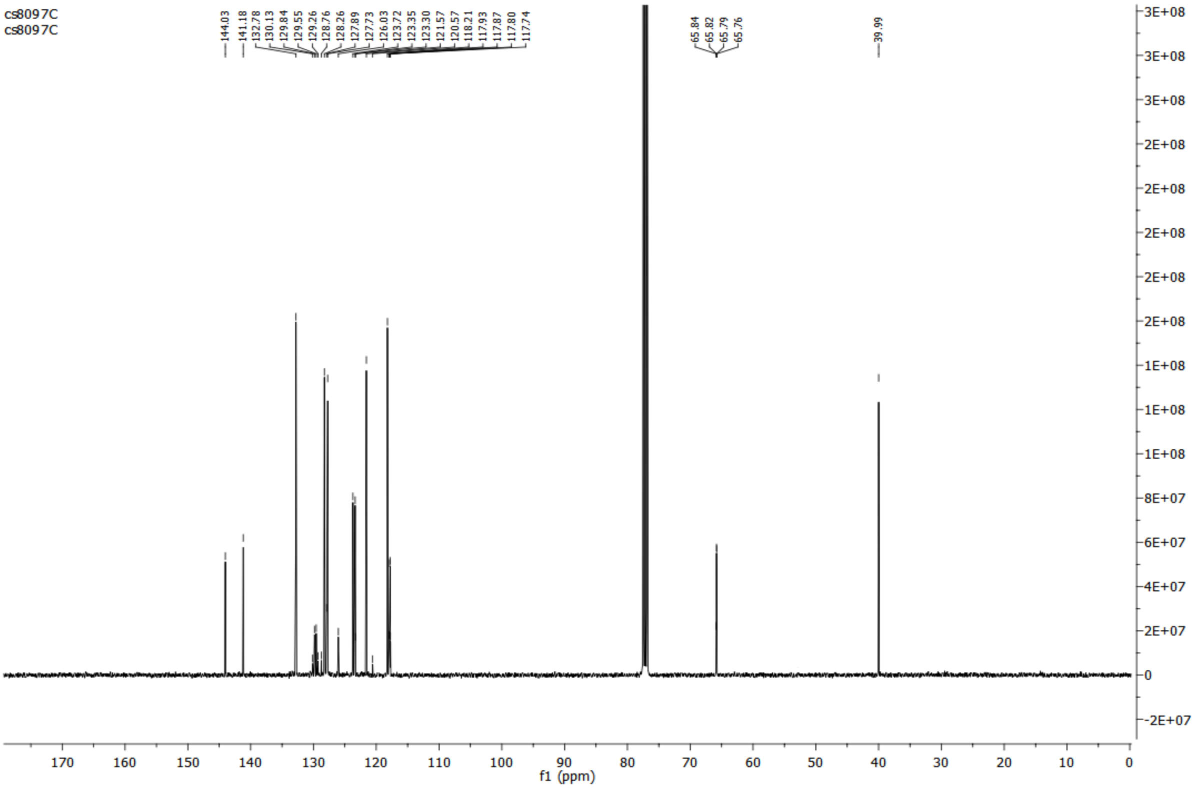

8-(trifluoromethyl)-10,11-dihydro-*5H*-dibenzo[*b*,*f*]azepin-10-ol **4e**

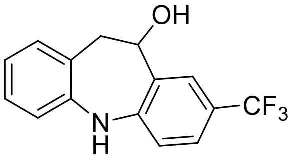

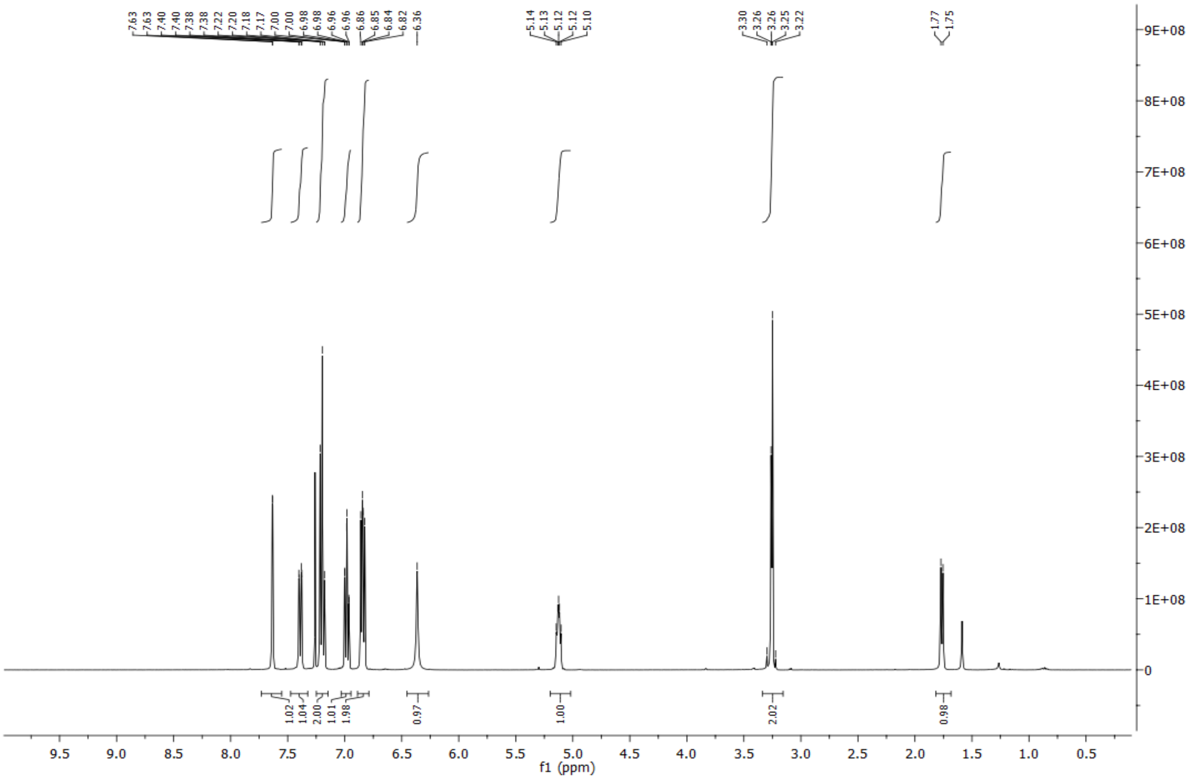

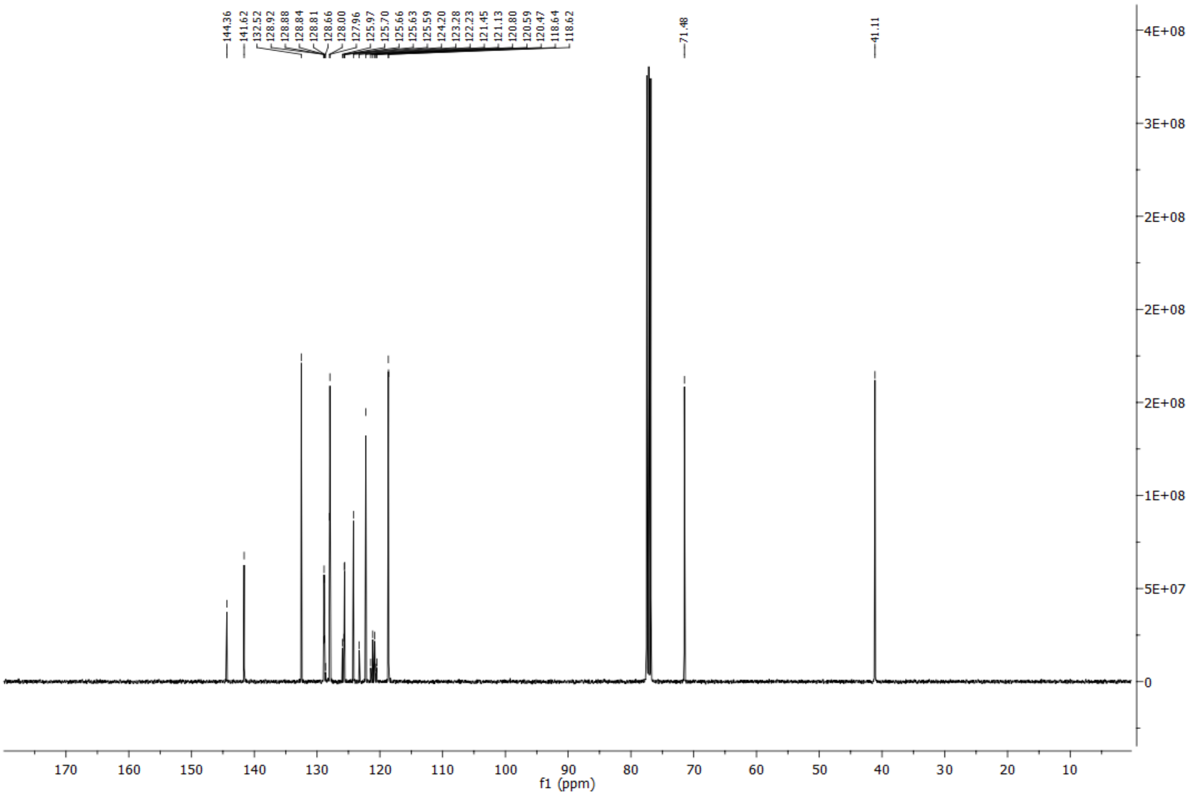

7-(trifluoromethyl)-10,11-dihydro-*5H*-dibenzo[*b*,*f*]azepin-10-ol **4f**

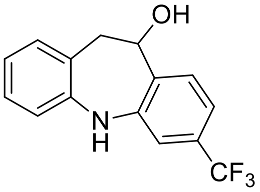

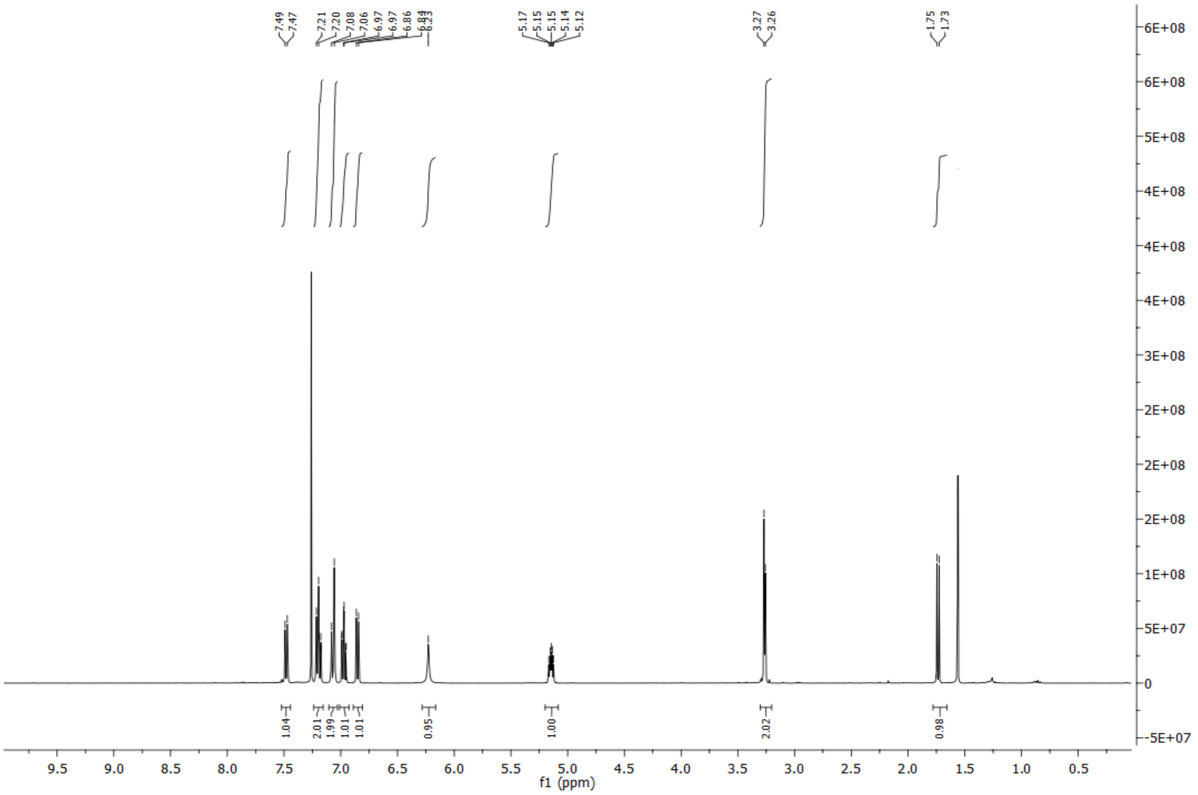

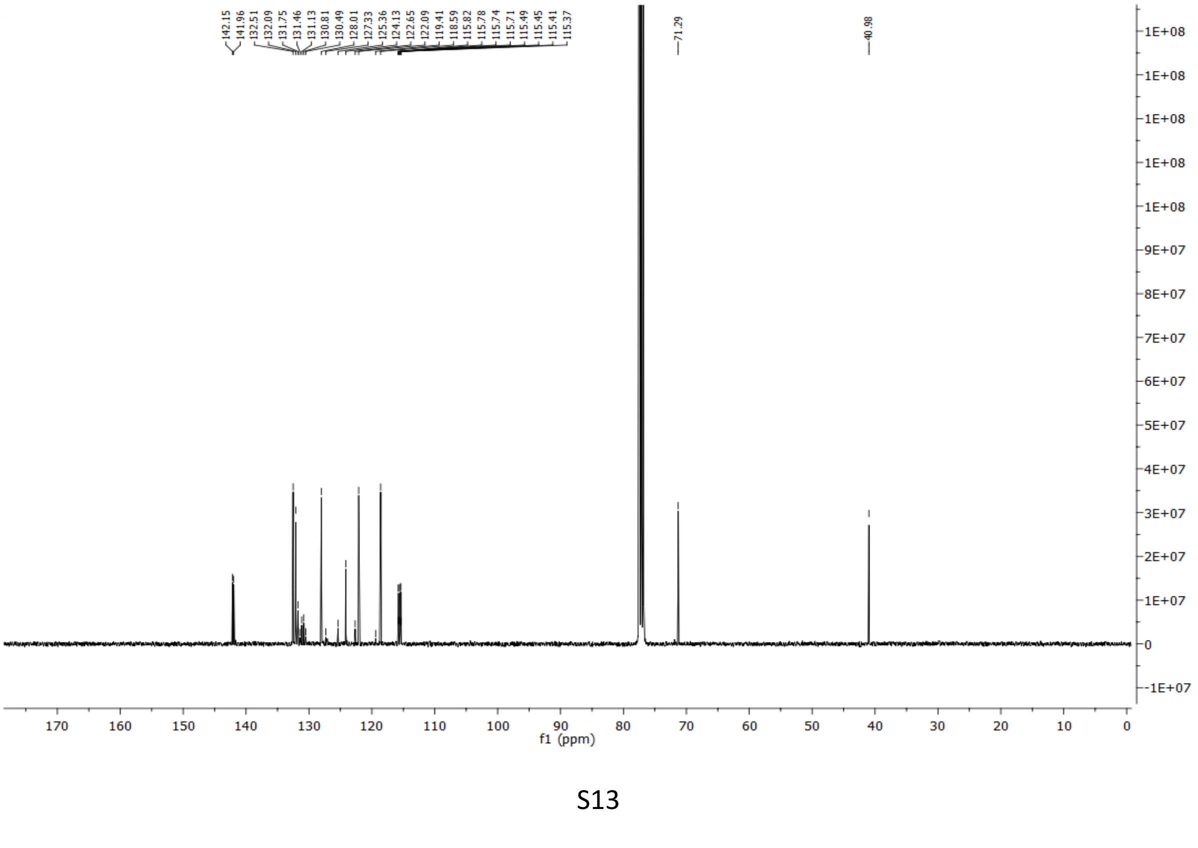

6-(trifluoromethyl)-10,11-dihydro-*5H*-dibenzo[*b*,*f*]azepin-10-ol **4g**

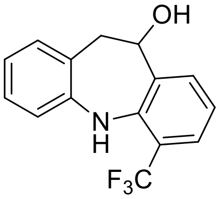

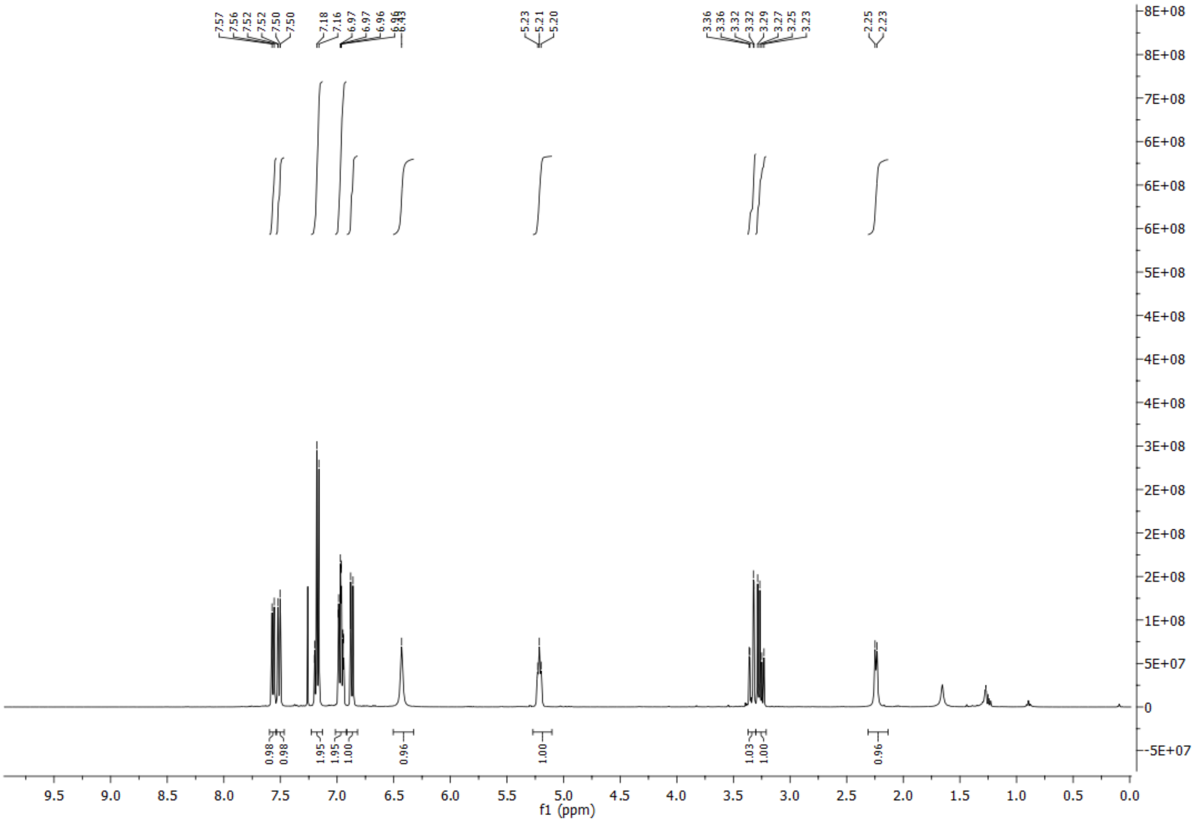

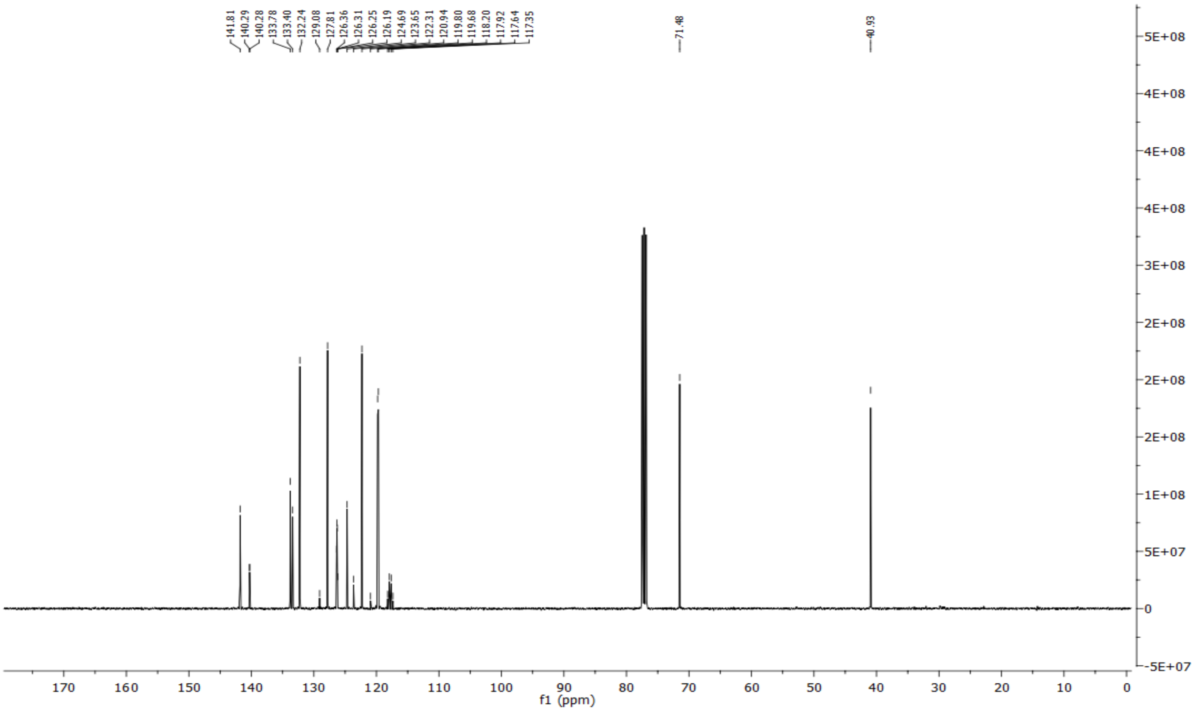

9-methyl-10,11-dihydro-*5H*-dibenzo[*b*,*f*]azepin-10-ol **4h**

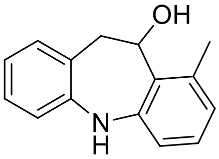

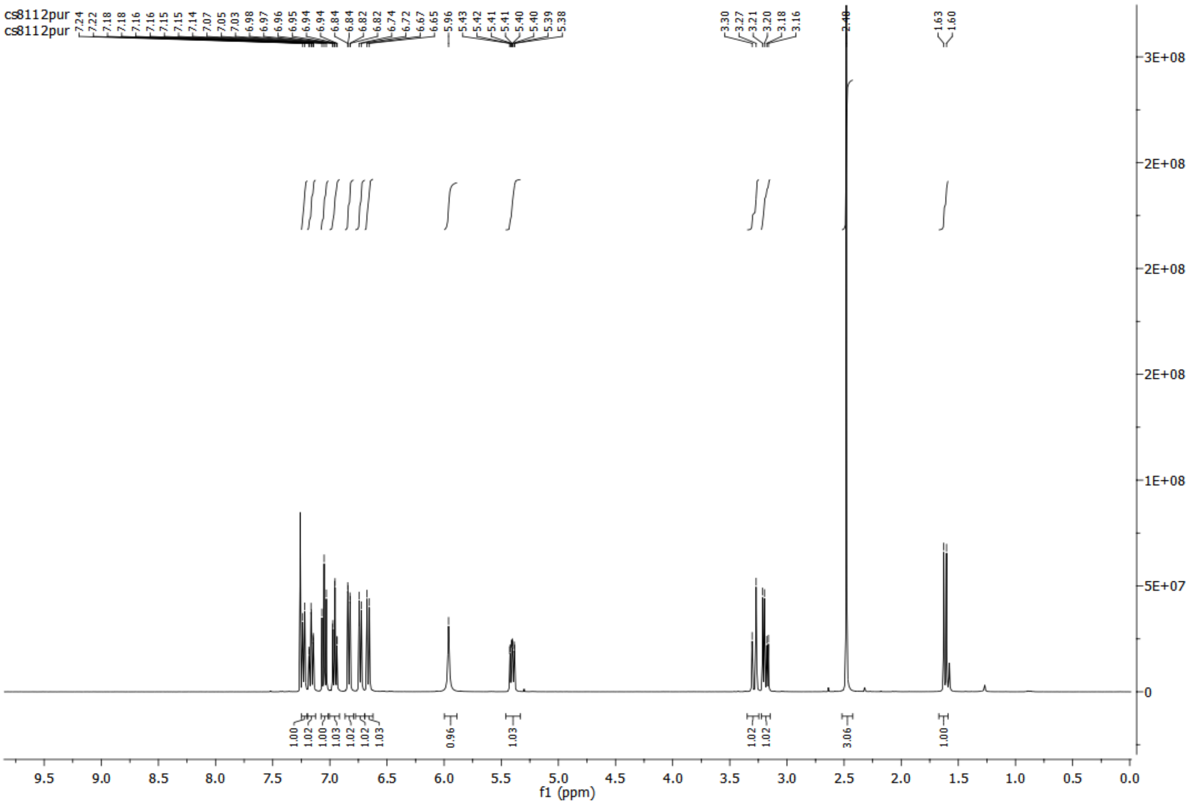

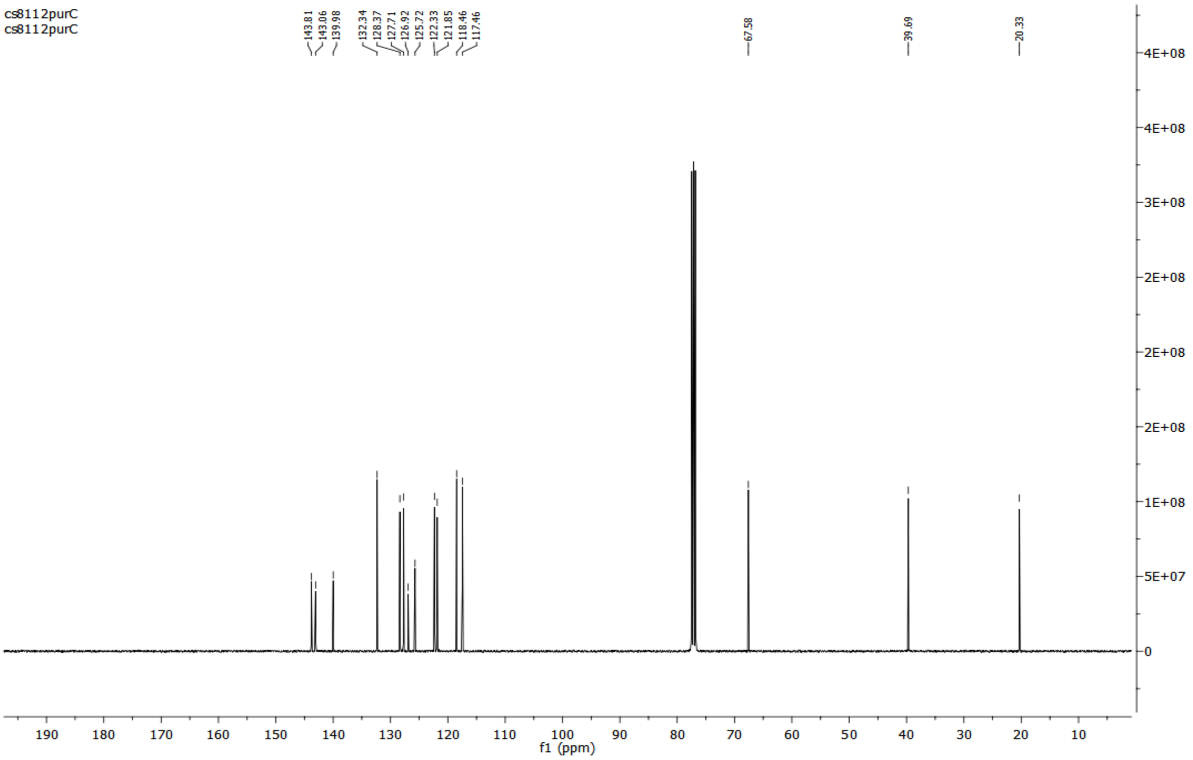

2-methyl-10,11-dihydro-*5H*-dibenzo[*b*,*f*]azepin-10-ol **4i**

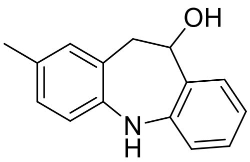

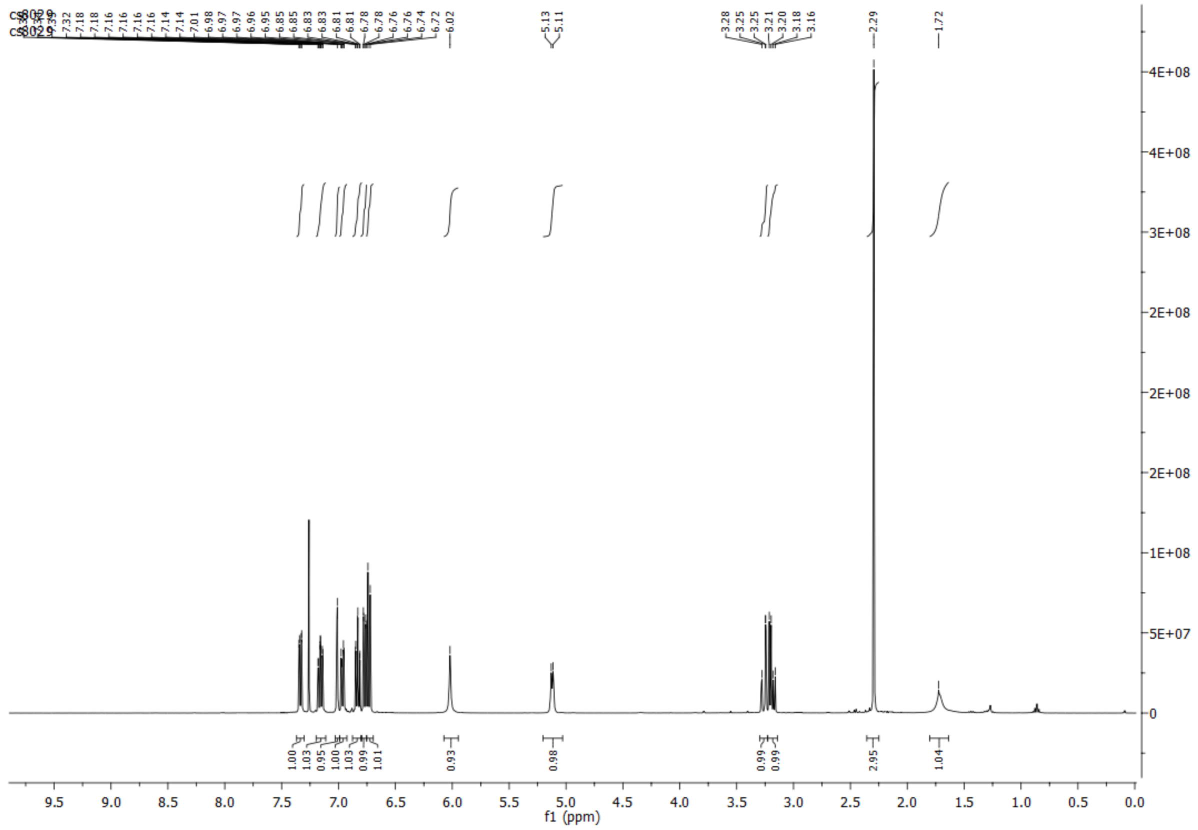

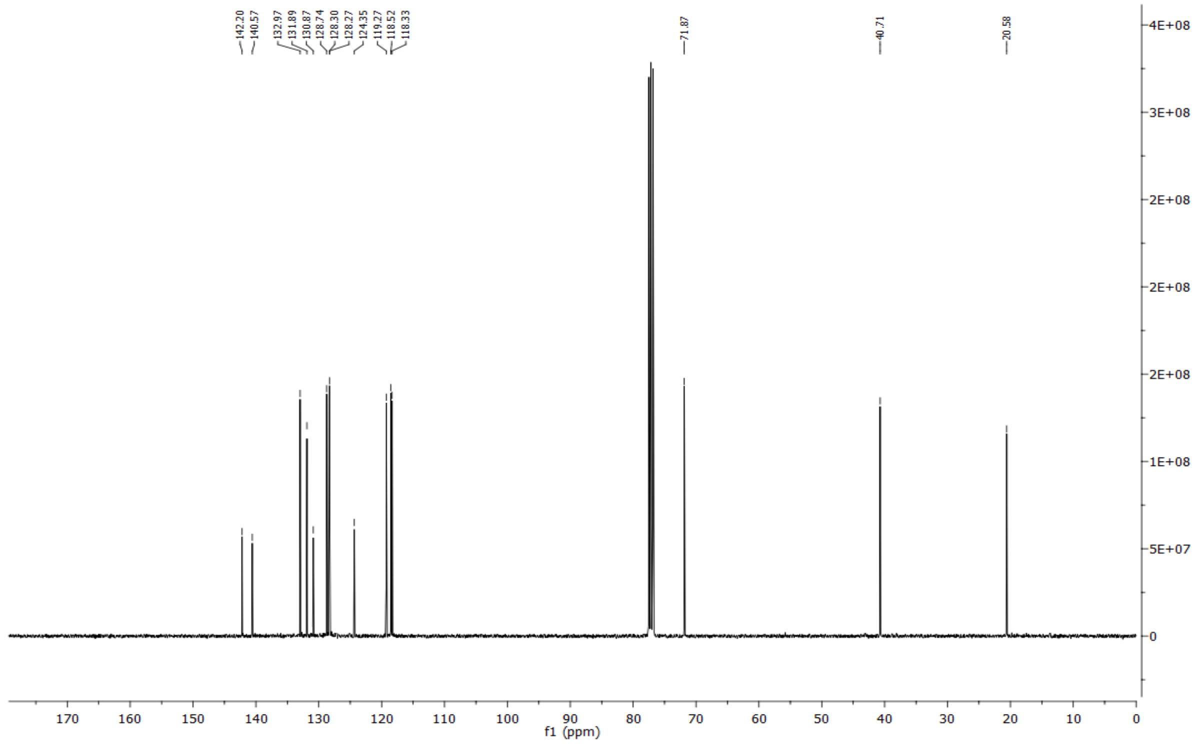

2-methyl-8-(trifluoromethyl)-10,11-dihydro-*5H*-dibenzo[*b*,*f*]azepin-10-ol **4j**

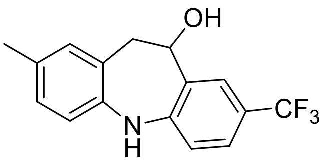

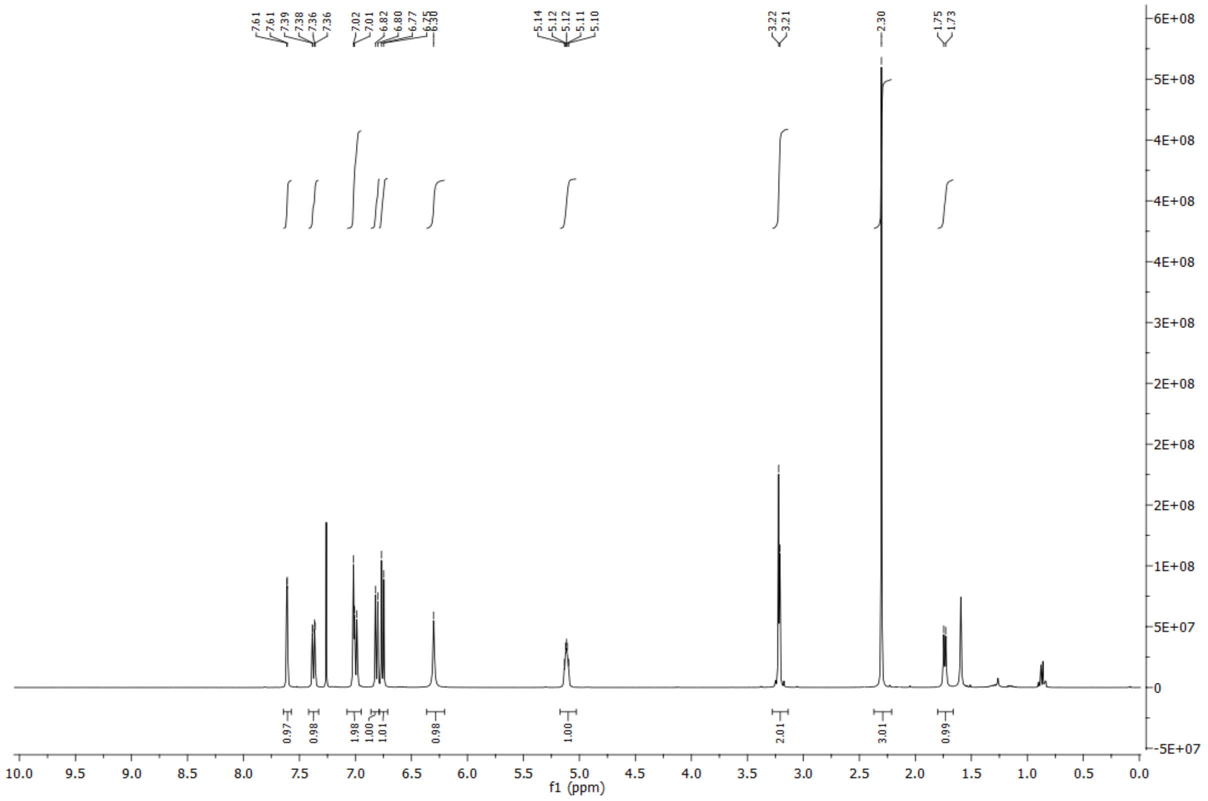

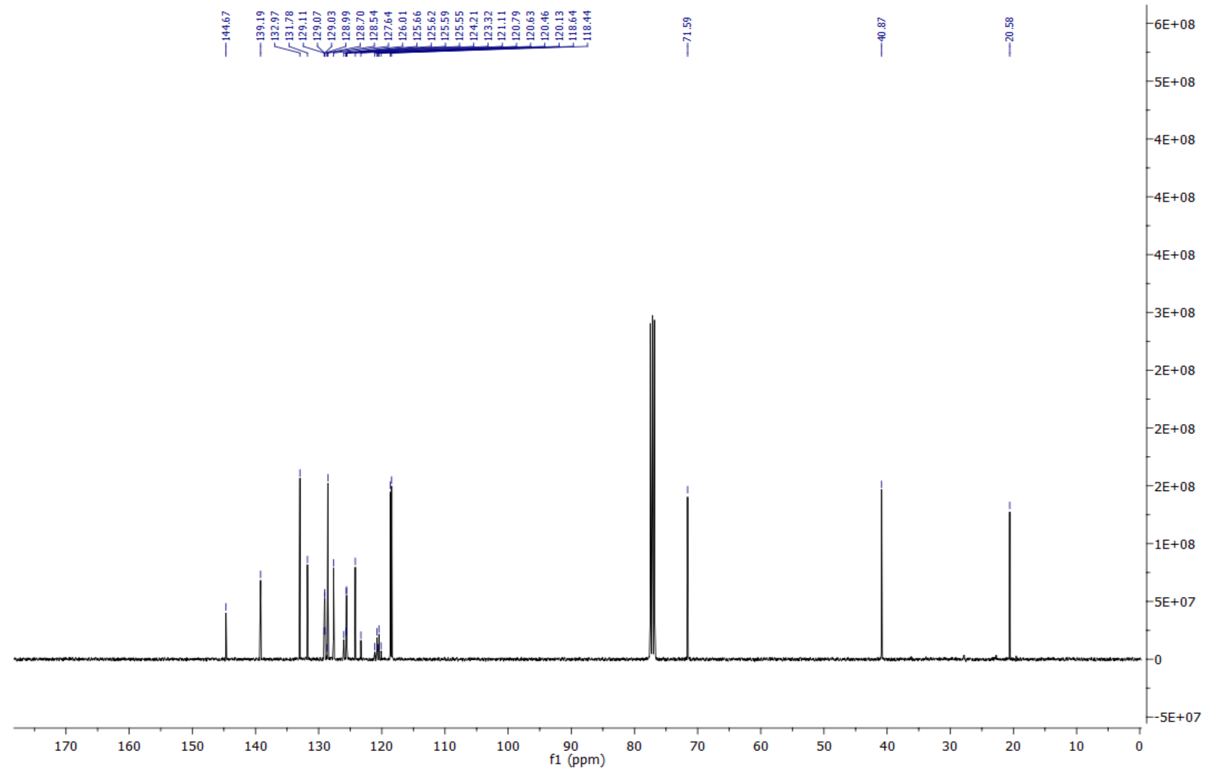

2-chloro-8-(trifluoromethyl)-10,11-dihydro-*5H*-dibenzo[*b*,*f*]azepin-10-ol **4k**

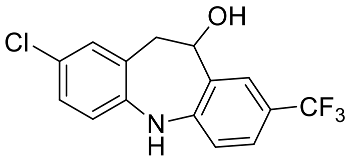

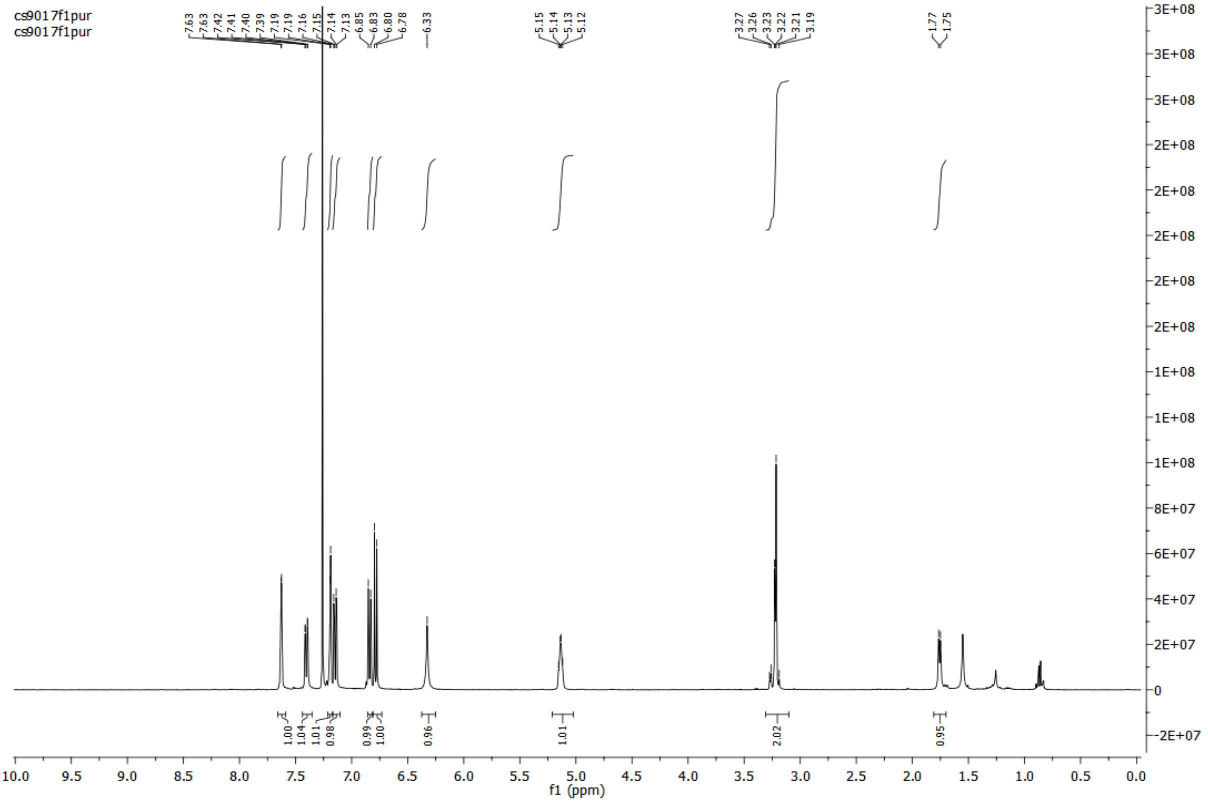

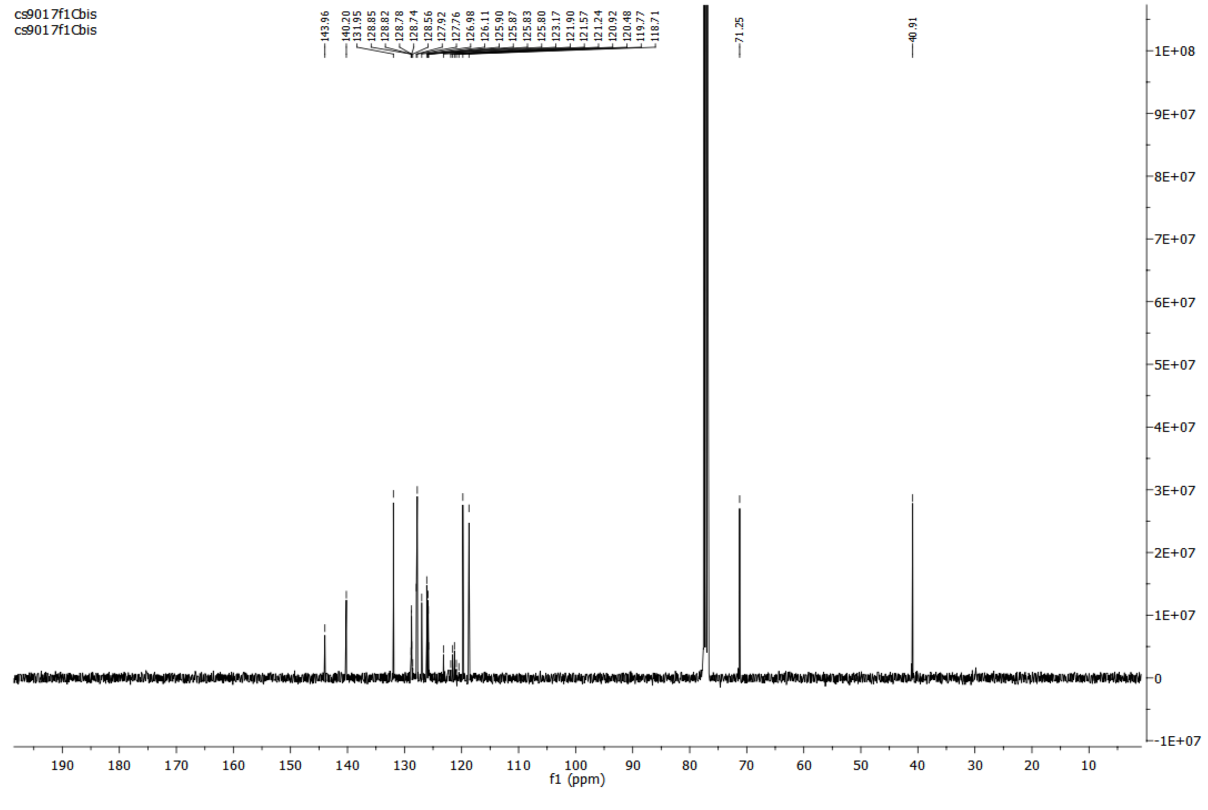

1-chloro-6-(trifluoromethyl)-10,11-dihydro-*5H*-dibenzo[*b*,*f*]azepin-10-ol **4l**

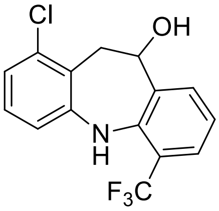

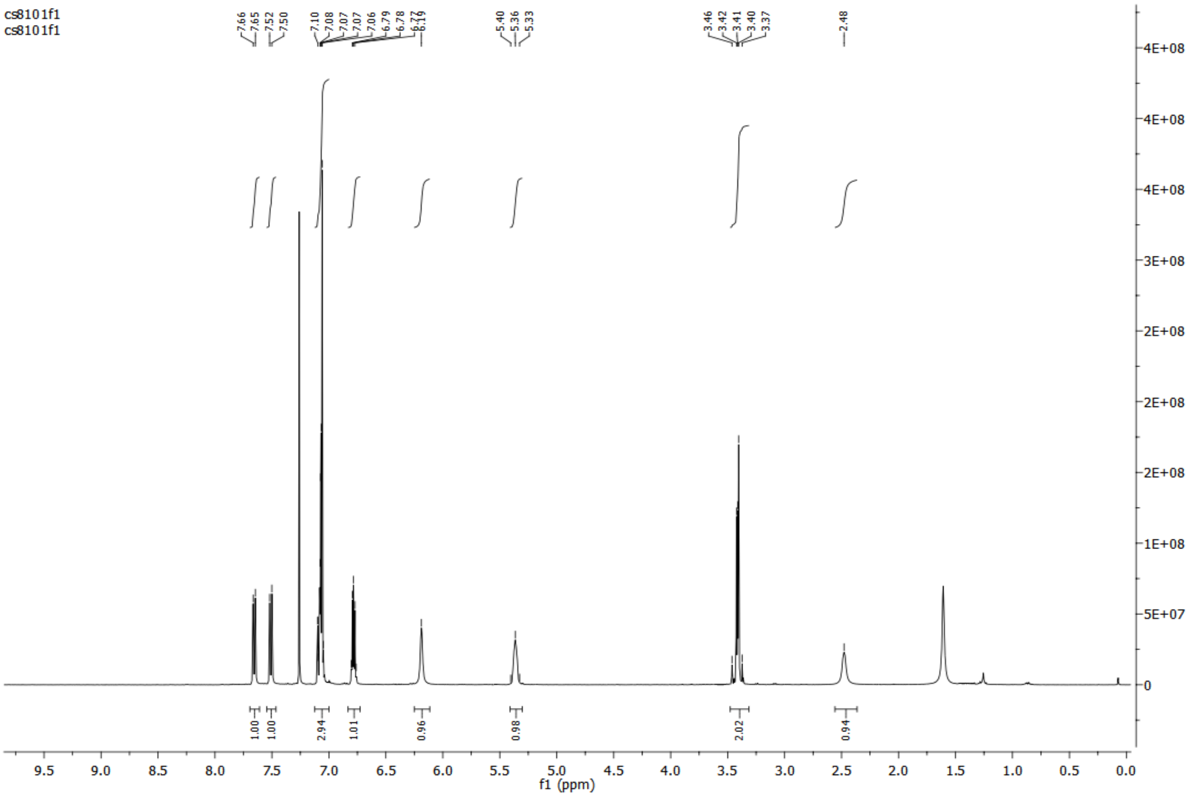

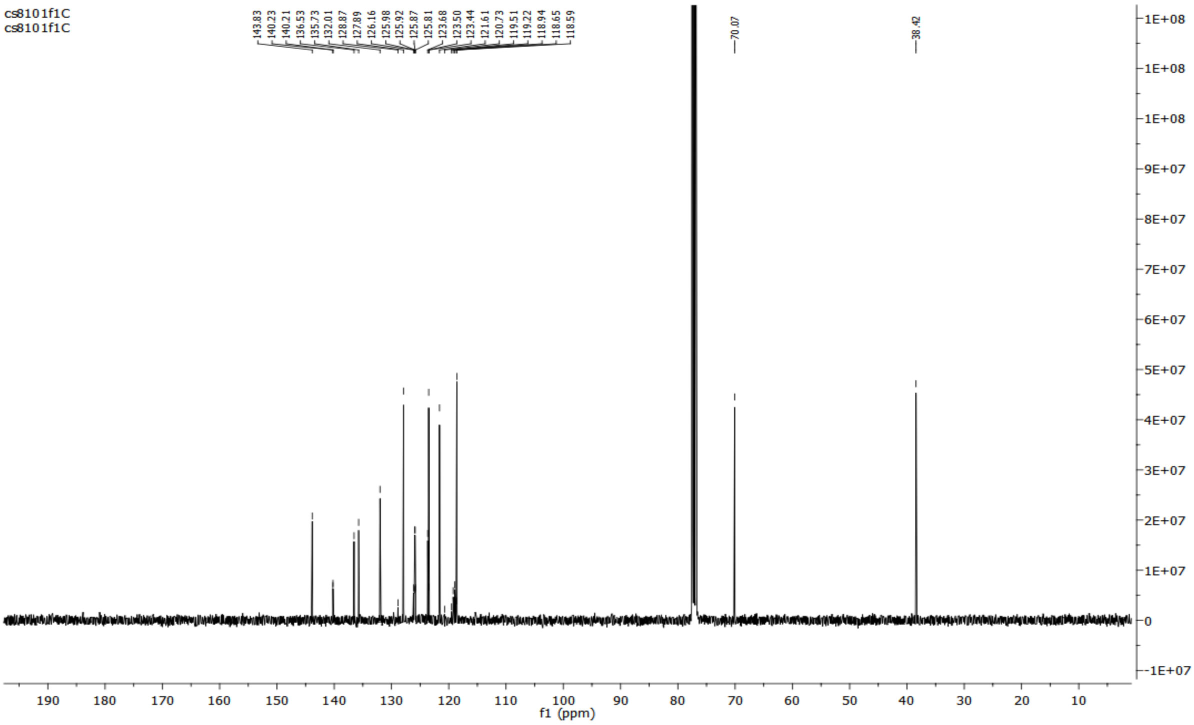

Supplement: RA-015-D5RA00909J-s001 [file RA-015-D5RA00909J-s001.pdf]
